# Genome-wide analysis identifies colonic genes differentially associated with serum leptin and insulin concentrations in C57BL/6J mice fed a high-fat diet

**DOI:** 10.1371/journal.pone.0171664

**Published:** 2017-02-07

**Authors:** Sung-Eun Kim, Jinsil Choo, Joon Yoon, Jae Ryang Chu, Yun Jung Bae, Seungyeoun Lee, Taesung Park, Mi-Kyung Sung

**Affiliations:** 1 Department of Food and Nutrition, Sookmyung Women’s University, Seoul, Republic of Korea; 2 Department of Life Systems, Sookmyung Women’s University, Seoul, Republic of Korea; 3 Interdisciplinary Program in Bioinformatics, Seoul National University, Seoul, Republic of Korea; 4 Division of Food Science and Culinary Arts, Shinhan University, Gyeonggi-do, Republic of Korea; 5 Department of Mathematics and Statistics, Sejong University, Seoul, Republic of Korea; 6 Department of Statistics, Seoul National University, Seoul, Republic of Korea; University of Hawai'i at Manoa College of Tropical Agriculture and Human Resources, UNITED STATES

## Abstract

Obesity-induced chronic inflammation is known to increase the risk of ulcerative colitis, Crohn’s disease, and colorectal cancer. Accumulating evidence suggests that leptin and insulin are key molecules linking obesity with diseases of the lower intestine. Here, we identified serum phenotype-associated genes in the colon of diet-induced obese mice as early biomarkers of obesity-associated colonic diseases. C57BL/6J mice were fed with either normal diet (ND, 15% of fat calories) or high-fat diet (HFD, 45% of fat calories) for 8 weeks. Serum concentrations of insulin, insulin-like growth factor-1 (IGF-1), leptin, and adiponectin were measured as obesity-related phenotypic markers. Genome-wide gene expression profiles of colon tissue were determined, followed by statistical analyses to detect differentially expressed and serum phenotype-associated genes. HFD-fed mice showed higher serum concentrations of leptin (P < 0.001) and insulin (P < 0.01) than those in the ND group, whereas serum IGF-1 and adiponectin concentrations did not differ between the two dietary groups. Among differentially expressed genes affected by HFD, 135, 128, 110, and 341 genes were associated with serum levels of leptin, insulin, IGF-1, and adiponectin, respectively. We identified 17 leptin-associated genes and 4 insulin-associated genes that inversely responded to HFD and ND. Among these, leptin-associated *Peli3* (Pellino E3 ubiquitin protein ligase family member 3), *Creb1* (cAMP responsive element binding protein 1), and *Enpp2* (ectonucleotide pyrophosphatase/phosphodiesterase 2, autotaxin) and insulin-associated *Centg1* (AGAP2, ArfGAP with GTPase domain) are reported to play a role either in obesity or colonic diseases. mRNA expression of these genes was validated by RT-qPCR. Our data suggest *Peli3*, *Creb1*, *Enpp2*, and *Centg1* as potential early biomarker candidates for obesity-induced pathophysiological changes in the colon. Future studies verifying the function of these candidates are needed for the prevention, early detection, and treatment of colon diseases.

## Introduction

Obesity, a state of an excessive body fat, has emerged as an important public health issue due to the increased risk of abnormal intermediate conditions such as hyperinsulinemia, which contributes to the high prevalence of obesity-associated metabolic complications [1]. Growing evidence suggests that obesity-induced chronic inflammation has been closely associated with the risk of ulcerative colitis, Crohn’s disease, and colorectal cancer [2–4]. Obesity induced by a high-fat diet (HFD) exacerbates the inflammatory indications of ulcerative colitis [2] and the formation of malignant tumors in the colon [5]. A recent large prospective cohort study reports that the measures of obesity are associated with increased risk of Crohn’s disease [3]. Several epidemiological studies suggest a significant positive correlation between body mass index (BMI) and the risk of colorectal cancer [6, 7].

Evidence from animal studies also suggests that HFD-induced obesity results in higher concentrations of serum leptin, insulin, and insulin-like growth factor-1 (IGF-1), and facilitates colon tumor formation [5]. Leptin is known to contribute to the colon tumor development in genetic models of obesity [8]. Serum leptin levels are likely influenced by insulin and are known to enhance inflammatory immune responses [9, 10]. IGF-1 is involved in cell cycle progression and is highly expressed in colorectal tumor tissues, indicating that IGF-1 participates in colon carcinogenesis [11]. Adiponectin is beneficial to inhibit colon tumor growth and improves insulin sensitivity by decreasing the serum insulin levels in mice fed with a HFD [12]. In addition, obesity-associated alterations including increased circulating concentrations of insulin, IGF-1, and leptin as well as decreased adiponectin levels, activate the phosphatidylinositol-3-kinase (PI3K)/Akt signaling pathway that promotes cell survival and proliferation, thus leading to colon carcinogenesis [13]. Thus, identifying the biomarkers associated with obesity-associated colonic diseases including colorectal cancer is important for the prevention, early detection, and treatment of these diseases.

Recent advances in genome-wide analyses have enabled interpretation of complex interactions induced by several genetic or phenotypic changes, and have therefore been used to determine patterns of gene expression associated with diseases and nutrition intervention [14]. Several studies have investigated the gene expression patterns of adipocytes and hepatic tissues in response to a HFD [15–17]. However, the effect of a HFD on colonic gene expression profiles is not clearly understood. In this study, we performed a microarray analysis to investigate the patterns of gene expression in colon tissue affected by HFD and further identified the serum phenotype-associated genes in the colon as early biomarker candidates of obesity-associated colonic diseases.

## Materials and methods

### Animals

Twenty 4-weeks-old male C57BL/6J mice (SLC Japan, Tokyo, Japan) were housed under controlled conditions of humidity (50 ± 5%), room temperature (23 ± 2°C), and light (12 h light/dark cycle). After a week-long acclimatization to a chow diet, the animals were randomly assigned to either normal diet (ND, 15% of fat calories) or high-fat diet (HFD, 45% of fat calories) groups (n = 10 per dietary group). The experimental diets were prepared according to the American Institute of Nutrition (AIN)-76A diet with modification of the fat sources (Table 1). The experimental diets and water were provided *ad libitum* for 8 weeks. All procedures were approved by the Institutional Animal Care and Use Com, Seoul, Komittee of Sookmyung Women’s University (SMU-IACUC-2011-0401-005). At necropsy, mice were anesthetized with a 2:1 mixture of Zoletil (Virbac, Magny-en-Vexin, France) and Rompun (Bayer, Seoul, Republic of Korea) by intraperitoneal injection and blood and tissue samples were collected. Serum was separated by centrifuging whole blood at 650 × *g* for 20 min and stored at -80°C until analysis. Colon samples were stored at -80°C until used for microarray analysis.

**Table 1:** Composition of the experimental diets (g/kg diet)^a^.

Ingredients	Normal diet	High-fat diet
**Casein**	202.97	245.32
**DL-methionine**	3.12	3.68
**Corn starch**	145.23	93.25
**Sucrose**	483.87	310.77
**Fiber**	50.00	50.00
**Corn oil**	50.00	50.00
**Lard**	16.70	189.00
**Mineral mixture**^b^	35.80	43.17
**Vitamin mixture**^b^	10.30	12.33
**Choline bitartrate**	2.01	2.47
***tert*-Butyhydeoquinone**^c^	0.01	0.01
**Total**	1,000	1,000

^a^ Diets were prepared according to the AIN-76A diet.

^b^ Mineral and vitamin mixtures were prepared according to the AIN-76A diet.

^c^ Antioxidant agent:0.01 g/50 g lipids.

### Serum phenotype measurements

Serum concentrations of leptin, insulin, IGF-1, and adiponectin were determined using enzyme-linked immunosorbent assay (ELISA) kits according to the manufacturer’s protocols (leptin, R&D Ann Arbor, MI, USA; insulin, Linco-Millipore Corp., MA, USA; IGF-1, R&D Ann Arbor, MI, USA; and adiponectin, Biovendor, Brno, Czech Republic).

### Microarray analysis

Genome-wide gene expression profiles of the colon was analyzed using microarray to compare differentially expressed genes in response to the ND and HFD diets at 8 week. Total RNA was isolated from the colon tissue of each mouse using Trizol^®^ (Invitrogen Life Technologies, Carlsbad, CA, USA) and was purified using RNeasy columns (Qiagen, Valencia, CA, USA) according to the manufacturers’ protocols. After processing with DNase digestion, RNA purity and integrity were evaluated by denaturing gel electrophoresis, OD 260/280 ratio, and analyzed on the Agilent 2100 Bioanalyzer (Agilent Technologies, Palo Alto, CA, USA). Total RNA was amplified and purified using the Illumina^®^ TotalPrep^™^ RNA amplification kit (Ambion, Austin, TX, USA) to yield biotinylated cRNA according to the manufacturer’s protocol. The biotinylated cRNA generated from the colon tissue samples was hybridized onto the Illumina MouseWG-6 v2 Expression BeadChip (Illumina, Inc., San Diego, CA, USA) targeting over 45,200 annotated RefSeq transcripts. The BeadChip was incubated for 16–18 h at 58°C according to the manufacturer's instructions. After washing and staining, each BeadChip was scanned using the Illumina Bead Array Reader Confocal Scanner according to the manufacturer's instructions. Raw data were extracted using GenomeStudio^®^ version 2009.2 (Illumina, Gene Expression Module version 1.5.4). The probe signal value was log-transformed and quantile normalized. All the data analyses and visualization of differentially expressed genes were conducted using R statistical language v. 2.4.1. For comparison between two dietary groups, an independent *t*-test was performed (our data are not repeatedly measured). Genes with a *t*-test P-value < 0.05 and a log_2_ fold change of ≥ 0.5849 (fold change ≥ 1.5) or ≤ -0.5849 (fold change ≤ 0.66) were selected and further analyzed using the Database for Annotation, Visualization, and Integrated Discovery (DAVID, http://david.abcc.ncifcrf.gov) for functional annotation analysis. The false discovery rate (FDR)-adjusted P-values of differentially expressed colonic genes in response to a HFD are presented in S1 Table. A less stringent threshold was applied for such screening due to sample size (n = 3 for the ND group; n = 6 for the HFD group). However, these statistically significant candidate genes were later technically validated through real-time quantitative polymerase chain reaction (RT-qPCR) to resolve false-positive issues.

### Data analysis for identification of serum phenotype-associated genes

In order to detect the differentially expressed genes (DEGs) and serum phenotype-associated genes (PAGs), the following four models were considered sequentially:

$${\text{M}}_{\text{DEG}}:\text{ Expression}=Group+e,\;e\sim N\left(0,\;\sigma^{2}I\right)$$

$${\text{M}}_{\text{PAG}1}:\text{ Phenotype}=Group+Expression+\left(Group*Expression\right)+e,\;e\sim N\left(0,\;\sigma^{2}I\right)$$

$${\text{M}}_{\text{PAG}2}:\text{ Phenotype}=Group+Expression+e,\;e\sim N\left(0,\;\sigma^{2}I\right)$$

$${\text{M}}_{\text{PAG}3}:\text{ Phenotype}=Expression+e,\;e\sim N\left(0,\;\sigma^{2}I\right)$$

[Separately for each group]

M_DEG_ is for DEG detection using the *t*-test, and the remaining are F-tests for PAG detection. In particular, M_PAG1_ tests for the significance of slope difference between the ND and HFD groups. M_PAG2_ detects the genes that have differential expression, yet have a common slope for the ND and HFD groups (genes with insignificant P-value for the interaction term). M_PAG3_ is used when the interaction term of M_PAG1_ is significant. The effect of gene expression on the phenotype changes with the level for the group, and models with fixed group variables should be fitted accordingly. In this study, we focused on identification of genes that were detected as significant both in M_DEG_ (P < 0.05) and in M_PAG1_ (interaction P-value < 0.05), with opposite slope for the ND and HFD groups. We provided the FDR-adjusted P-values of all the genes in S2–S5 Tables.

### Real-time quantitative polymerase chain reaction analysis

Template RNA isolated from the colon tissue was reverse-transcribed using a cDNA Synthesis kit (PhileKorea Technology, Seoul, Republic of Korea) according to manufacturer’s protocol. RT-qPCR was performed on a 7500 Fast Real Time PCR system (Applied Biosystems, Foster City, CA, USA) using a QuantiMix SYBR kit (PhileKorea Technology, Seoul, Republic of Korea). The primer sequences were as follows: *Peli3* (forward: TGAGATCTCGATCCCAGGCA; reverse: AGGGCACAACCAGTCTAAGC); *Creb1* (forward: CACAGACCACTGATGGACAGCA; reverse: AGGACGCCATAACAACTCCAGG); *Enpp2* (forward: CTATCCTTCAGTGGCTTTCCC; reverse: GTCTTGTCAATCTCCCTCAGAG); *Centg1* (forward: CGTGAGGAATCTATGGATGAGG; reverse: CCCTAAGGCTGTGGTTAATAGC); and GAPDH (forward: TGTGTCCGTCGTGGATCTGA; reverse: CCTGCTTCACCACCTTCTTG). The reaction conditions were 10 min at 95°C followed by 40 cycles of 15 s at 95°C and 1 min at 60°C. All signals were normalized to the GAPDH mRNA levels as the housekeeping gene. Relative gene expression data was analyzed using the comparative threshold (C_t_) method [18].

### Statistical analysis

Differences in final body weight, tissue weight, and serum measurements between the two dietary groups were analyzed by the Student’s *t*-test using SAS 9.4 (SAS Institute, Inc., Cary, NC, USA). The results with a two-tailed P-value < 0.05 were considered statistically significant.

## Results

### Body weight, tissue weights, and serum phenotype concentrations

The food intake did not significantly differ between the two dietary groups, whereas the final body weight of HFD-fed mice was greater than that of the ND group (P < 0.001) (Table 2). The weights of the liver and total white adipose tissues were also increased in the HFD group compared with those in the ND group (P < 0.01) (Table 2). Mice fed HFD showed higher serum concentrations of leptin (P < 0.001) and insulin (P < 0.01) compared with those in the ND group, whereas serum IGF-1 and adiponectin concentrations did not differ between the two groups (Table 2).

**Table 2:** Final body weight, tissue weight, and serum phenotypes in high-fat diet fed C57BL/6J mice.

	Normal diet	High-fat diet
**Food intake (g/day)**	2.66 ± 0.10	2.83 ± 0.07
**Final body weight**	28.58 ± 0.42	33.59 ± 1.04**
**Liver weight**	1.00 ± 0.03	1.20 ± 0.05*
**Total white adipose tissues weight (% of body weight)**	2.77 ± 0.25	5.14 ± 0.35**
**Serum phenotypes levels**		
** Leptin (ng/mL)**	6.48 ± 1.21	31.56 ± 3.56**
** Insulin (ng/mL)**	0.21 ± 0.02	0.59 ± 0.10*
** IGF-1 (ng/mL)**	283.16 ± 16.32	294.39 ± 13.16
** Adiponectin (μg/mL)**	13.19 ± 2.49	10.76 ± 1.25

Data are presented as mean ± SEM (n = 10 per group);

* P < 0.01,

** P < 0.001, statistically significant compared with the normal diet.

### Effect of high-fat diet on genome-wide gene expression profiles in colon tissue

**Fig 1:**
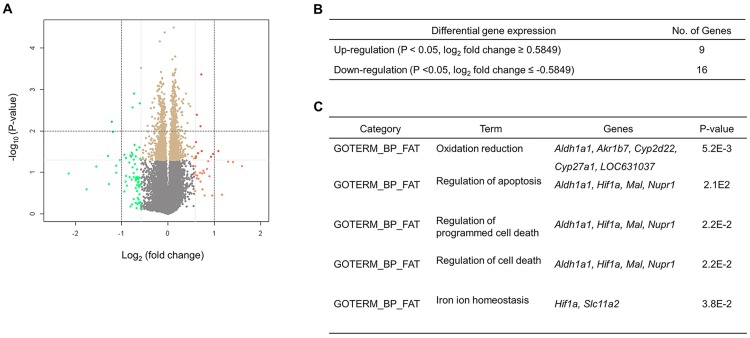
Volcano plot and the number and biological processes associated with differentially expressed genes in the colon tissue of high-fat diet fed C57BL/6J mice. The volcano plot represents the relationship between the fold change and significance between the normal diet and high-fat diet groups; the x-axis indicates the difference in gene expression between two dietary groups as log_2_ of their fold change, and the y-axis indicates the negative log_10_ of P-values for the *t*-test (**A**). Twenty-five genes with a *t*-test P-value < 0.05 and a log_2_ fold change of ≥ 0.5849 (fold change ≥ 1.5) or ≤ -0.5849 (fold change ≤ 0.66) were selected and further analyzed using the Database for Annotation, Visualization, and Integrated Discovery (DAVID) for functional annotation analysis (**B**, **C**).

The volcano plot displays the relationship between fold change and significance between the ND and HFD groups as shown in Fig 1A. We identified 9 upregulated and 16 downregulated genes with t-test P-values < 0.05 and log_2_ fold changes of ≥ 0.5849 (fold change ≥ 1.5) or ≤ -0.5849 (fold change ≤ 0.66) affected by the HFD (Fig 1B; Table 3). The biological processes of these DEGs included oxidation-reduction, regulation of apoptosis/programmed cell death, and regulation of cell death (Fig 1C).

**Table 3:** List of differentially expressed genes in the colon tissue of high-fat diet fed C57BL/6J mice.

	Accession	Symbol	Definition	Log_2_ fold change	P-value
**Up-regulation**	XM_001474081.1	LOC383196*	PREDICTED: Mus musculus hypothetical LOC383196 (LOC383196), mRNA.	1.0886	0.0305
	NM_001039562.1	*Ankrd37*	Mus musculus ankyrin repeat domain 37 (Ankrd37), mRNA.	0.9776	0.0361
	NM_009477.1	Upp1*	Mus musculus uridine phosphorylase 1 (Upp1), mRNA.	0.7288	0.0306
	NM_009264.2	*Sprr1a*	Mus musculus small proline-rich protein 1A (Sprr1a), mRNA.	0.7181	0.0004
	XM_135511	*9030605I04Rik*		0.7021	0.0078
	AK083478	*Slc11a2*		0.6506	0.0340
	NM_010431.1	*Hif1a*	Mus musculus hypoxia inducible factor 1, alpha subunit (Hif1a), mRNA.	0.6204	0.0041
	NM_174865.1	*Klk15*	Mus musculus kallikrein related-peptidase 15 (Klk15), mRNA.	0.6053	0.0186
	XR_031459.1	*LOC100045250*	PREDICTED: Mus musculus hypothetical protein LOC100045250 (LOC100045250), misc RNA.	0.6007	0.0447
**Down-regulation**	XM_982144.1	*LOC631037*	PREDICTED: Mus musculus similar to CYP4B1 (LOC631037), mRNA.	-1.2916	0.0402
	NM_013467.3	*Aldh1a1*	Mus musculus aldehyde dehydrogenase family 1, subfamily A1 (Aldh1a1), mRNA.	-1.2109	0.0060
	NM_021456.2	*Ces1*	Mus musculus carboxylesterase 1 (Ces1), mRNA.	-1.1910	0.0105
	NM_010762.4	Mal*	Mus musculus myelin and lymphocyte protein, T-cell differentiation protein (Mal), mRNA.	-0.9453	0.0375
	NM_008926.3	*Prkg2*	Mus musculus protein kinase, cGMP-dependent, type II (Prkg2), mRNA.	-0.9098	0.0451
	NM_033603.2	*Amn*	Mus musculus amnionless (Amn), mRNA.	-0.8076	0.0389
	NM_025655.2	*Tmigd1*	Mus musculus transmembrane and immunoglobulin domain containing 1 (Tmigd1), mRNA.	-0.7966	0.0423
	NM_173404.2	*Bmp3*	Mus musculus bone morphogenetic protein 3 (Bmp3), mRNA.	-0.7821	0.0337
	NM_026183.2	*1300013J15Rik*	Mus musculus RIKEN cDNA 1300013J15 gene (1300013J15Rik), mRNA.	-0.7698	0.0370
	NM_019823.3	*Cyp2d22*	Mus musculus cytochrome P450, family 2, subfamily d, polypeptide 22 (Cyp2d22), mRNA.	-0.7684	0.0027
	XM_127434	*9030624O13Rik*		-0.7392	0.0491
	NM_009731.1	*Akr1b7*	Mus musculus aldo-keto reductase family 1, member B7 (Akr1b7), mRNA.	-0.7330	0.0013
	NM_175535.3	Arhgap20*	Mus musculus Rho GTPase activating protein 20 (Arhgap20), mRNA.	-0.6796	0.0287
	NM_026085.2	*3110049J23Rik*	Mus musculus RIKEN cDNA 3110049J23 gene (3110049J23Rik), mRNA.	-0.6182	0.0249
	NM_019738.1	*Nupr1*	Mus musculus nuclear protein 1 (Nupr1), mRNA.	-0.6095	0.0021
	NM_024264.3	*Cyp27a1*	Mus musculus cytochrome P450, family 27, subfamily a, polypeptide 1 (Cyp27a1), mRNA.	-0.5973	0.0467

Differentially expressed genes showed a t-test P-value < 0.05 and a log_2_ fold change of ≥ 0.5849 (fold change ≥ 1.5) or ≤ -0.5849 (fold change ≤ 0.66) based on the high-fat diet vs. normal diet comparison;

* A given gene is represented in the microarray set with multiple identifiers.

### Identification of serum phenotype-associated genes among the differentially expressed colonic genes in response to high-fat diet

**Fig 2:**
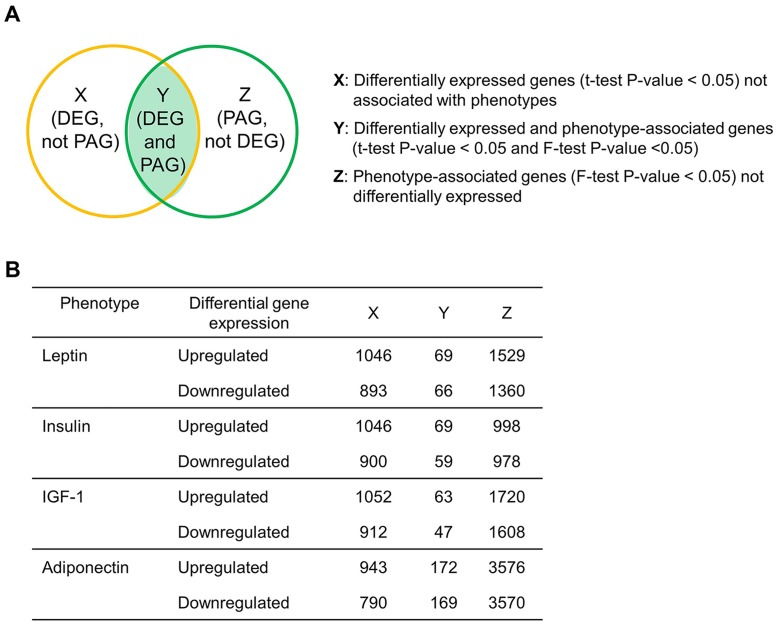
Scheme of bioinformatics analyses (A) and the number of differentially expressed genes and serum phenotype-associated genes in the colon tissue of high-fat diet fed C57BL/6J mice (B). DEG, differentially expressed genes; PAG, phenotype-associated genes.

We identified both the DEGs (*t*-test P-value < 0.05) and PAGs (F-test P-value < 0.05) in the colon tissue of HFD-fed mice based on four linear regression models as described previously (Fig 2A). The number of DEGs and PAGs is presented in Fig 2B. Among the differentially expressed genes, 135 and 128 genes were associated with serum concentrations of leptin and insulin, respectively (Fig 2B; S2 and S3 Tables). We also identified 110 and 341 genes related with serum IGF-1 and adiponectin concentrations, respectively (Fig 2B; S4 and S5 Tables).

We next focused on both the DEGs and PAGs (Y in Fig 2A) having an inverse relationship with the serum phenotypes, by the two different dietary types (P-value for interaction term (gene expression * diet groups) < 0.05). We identified 17, 4, 44, and 338 DEGs representing inverse associations with the serum levels of leptin, insulin, IGF-1, and adiponectin, respectively, depending on dietary fat content (S2–S5 Tables).

We further investigated serum leptin- and inulin-associated genes since these two phenotypes showed significant differences between the ND and HFD groups, as observed in the present study. The DEGs showing an inverse relationship with serum leptin by the two different dietary types included *Spnb1*, *Creb1*, *Myh7*, *Olfr1408*, *Enpp2*, *scl0002720*.*1_68*, *2610029G23Rik*, *Hddc2*, *LOC384276*, *E530015N03Rik*, *LOC676420*, *Kcnip3*, *Peli3*, *Kcnma1*, *Timm50*, *EG632964*, and *Slc18a1* (Table 4; S2 Table). The differentially expressed and serum insulin-associated genes with an inverse association depending on the dietary types consisted of *Centg1*, *4921515J06Rik*, *Spnb1*, and *scl0002720*.*1_68* (Table 4; S3 Table). *Spnb1* (spectrin beta, erythrocytic) and *scl0002720*.*1_68* were identified as both serum leptin- and insulin-associated genes; however, the functions of these genes are largely unknown.

**Table 4:** List of differentially expressed genes having inverse relationships with either serum leptin or serum insulin by the normal and high-fat diets.

Phenotype (No. of genes)	Gene	Accession	Definition	P-value*
**Leptin (17)**	**Upregulated**			
	*Myh7*	NM_080728.2	Myosin, heavy chain 7, cardiac muscle, beta	0.010
	*scl0002720*.*1_68*	AK053156.1	-	0.026
	*LOC384276*	XM_357535.1	-	0.027
	*E530015N03Rik*	XM_001476869.1	Anks1b, ankyrin repeat and sterile alpha motif domain containing 1B	0.027
	*Hddc2*	NM_027168.2	HD domain containing 2	0.027
	*Kcnip3*	NM_019789.2	Kv channel interacting protein 3, calsenilin	0.030
	*Peli3*	NM_172835.2	Pellino E3 ubiquitin protein ligase family member 3	0.031
	*EG632964*	XM_907275.3	Gm7098	0.041
	*Slc18a1*	NM_153054.2	Solute carrier family 18 member 1	0.048
	**Downregulated**			
	*Spnb1*	NM_013675.3	Spectrin beta, erythrocytic	0.002
	*Creb1*	NM_133828.2	cAMP responsive element binding protein 1	0.003
	*Olfr1408*	NM_146764	Olfactory receptor 1408	0.013
	*Enpp2*	AK038940	Ectonucleotide pyrophosphatase/phosphodiesterase 2, autotaxin	0.020
	*2610029G23Rik*	NM_026312.4	Pbdc1, Polysaccharide biosynthesis domain containing 1	0.026
	*LOC676420*	XR_031436.1	-	0.029
	*Kcnma1*	AK048773	Potassium channel, calcium activated large conductance subfamily M alpha member, 1	0.038
	*Timm50*	NM_025616.3	Translocase of inner mitochondrial member 50 homolog	0.039
**Insulin (4)**	**Upregulated**			
	*Centg1*	NM_001033263.1	AGAP2, ArfGAP with GTPase domain	0.016
	*4921515J06Rik*	NM_025723.2	Henmt1 (MEN1, methyltransferase homolog 1), Arabidopsis	0.037
	*scl0002720*.*1_68*	AK053156.1	-	0.045
	**Downregulated**			
	*Spnb1*	NM_013675.3	Spectrin beta, erythrocytic	0.038

An asterisk indicates a P-value for the interaction term between the gene expression and the diet group.

**Fig 3:**
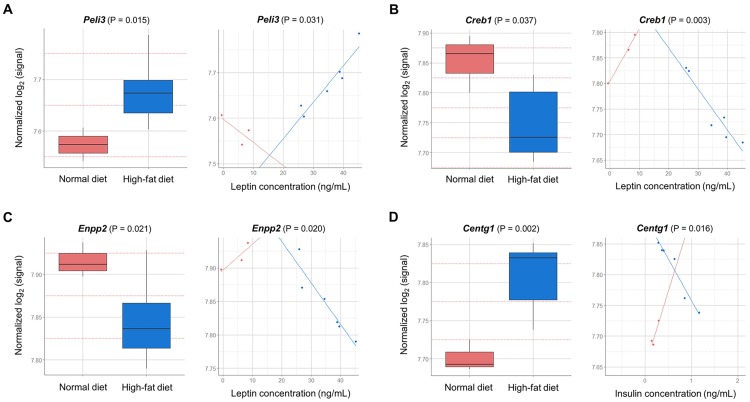
Representative differentially expressed genes having inverse relationships with serum leptin (A to C) and insulin (D) by diet type in C57BL/6J mice. The y-axis is gene expression level (log_2_ normalized) and the x-axis indicates the diet groups for the boxplots (left), and serum levels of the two phenotypes of interest for the line plots (right). The boxplot displays that there clearly is a difference between dietary groups, and the line plot displays the inverse relationship between the serum phenotypes and dietary groups; as an example, in panel A, the leptin level increases as expression level decreases for normal diet (negative slope), while expression level increases together with leptin level for high-fat diet (positive slope).

**Fig 4:**
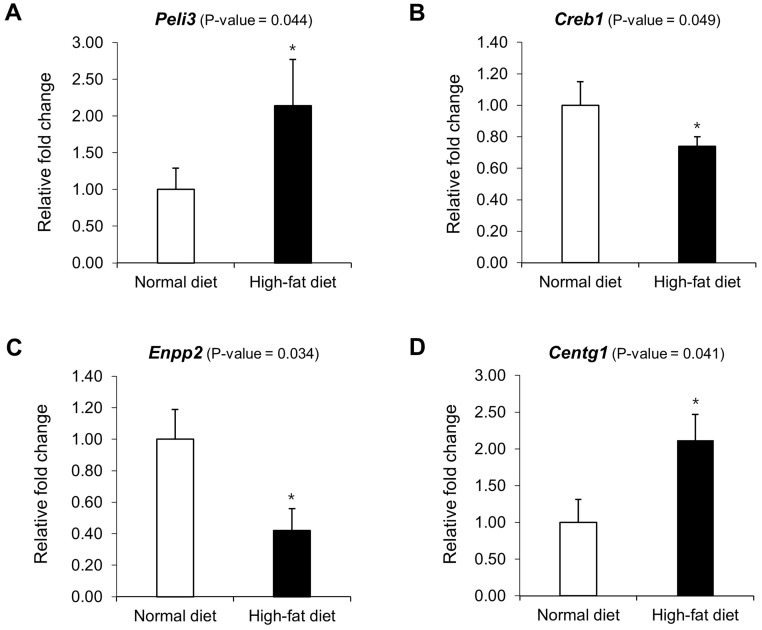
mRNA expression of genes associated with serum leptin and insulin concentrations in C57BL/6J mice. The expression of genes related with serum leptin (**A** to **C**) and insulin (**D**) concentrations was analyzed by RT-qPCR to validate the microarray-based expression results of these genes. The results were normalized to GAPDH mRNA expression. The data are expressed as mean ± SEM. *P < 0.05, statistically significant compared with the normal diet group.

Among these 21 genes, we selected *Peli3*, *Creb1*, *Enpp2*, and *Centg1* as these have been reported in previous studies to play a role in obesity or in colon-related diseases. As for serum leptin-associated genes, the relationship between serum leptin concentration and *Peli3* (Pellino E3 ubiquitin protein ligase family member 3) expression was positive in the HFD group, whereas a negative relationship was observed in the ND group (Fig 3). A negative relationship between serum leptin concentration and the expression of *Creb1* (cAMP responsive element binding protein 1) and *Enpp2* (ectonucleotide pyrophosphatase/phosphodiesterase 2, autotaxin) was observed in the HFD group, whereas, in the ND group, a positive relationship was observed (Fig 3). In addition, *Centg1* (AGAP2, ArfGAP with GTPase domain), one of the serum insulin-associated genes, was upregulated in the HFD group compared to that in the ND group with a significant interaction between the two dietary groups; the relationship between *Centg1* expression and serum insulin level was negative in the HFD group and positive in the ND group (Fig 3). We also confirmed the microarray-based expression results of the genes associated with serum leptin and insulin concentrations by RT-qPCR. The mRNA expression of all the genes was consistent with their expression in the microarray results (Figs 3 and 4), although the correlation between gene expression and protein expression is influenced by biological and technical factors [19].

## Discussion

In the present study, we determined the effect of a HFD on the genome-wide gene expression patterns in the colon and further identified DEGs having an inverse relationship with serum phenotypes by two different dietary types using statistical analyses, as early biomarker candidates of obesity-associated colonic diseases. Obesity induces abnormal intermediate conditions including hyperinsulinemia, hyperglycemia, and dyslipidemia, leading to an increased risk of type II diabetes, hypertension, cardiovascular diseases, and cancer [1]. Obesity-induced chronic inflammation is also known to increase the risk of ulcerative colitis, Crohn’s disease, and colorectal cancer [2–4].

We identified 9 upregulated and 16 downregulated genes in the colon tissue of HFD-fed mice. *Sprr1a* (small proline-rich protein 1A) was upregulated in response to HFD. Higher expression of *Sprr1a* is also observed both in the colonic mucosa of mice fed with a folate-deficient diet and in the normal tissue of patients with colorectal cancer compared to that of controls [20]. Several downregulated genes including *Mal*, *Prkg2*, *Amn*, *Tmigd1*, *9030624O13Rik*, and *Akr1b7* are known to be associated with colonic diseases. *Mal* (myelin and lymphocyte protein, T-cell differentiation protein) is a tumor-suppressor gene and a diagnostic biomarker for colorectal cancer due to its inactivation by promoter hypermethylation [21]. A recent study reported that *Mal* downregulation is associated with colorectal cancer metastasis [22]. Hypermethylation of the *Mal* promoter is also found in gastric cancers, suggesting that detection of *Mal* methylation could be a good prognostic marker for other types of cancer [23]. *Prkg2* (protein kinase, cGMP-dependent, type II) encodes cGMP-dependent protein kinase 2 (PKG2). cGMP signaling plays a critical role in intestinal homeostasis [24]. *Prkg2* knockout mice demonstrated crypt hyperplasia in the colon, and in the same study, ectopic *PKG2* was found to reduce colony formation and proliferation in colon cancer cells [25]. The expression of *Amn* (amnionless) is known to gradually decrease during the normal-adenoma-dysplasia-carcinoma transition, and is suggested as a potential biomarker for colon carcinogenesis [26]. In addition, the expression of *Tmigd1* (transmembrane and immunoglobulin domain containing 1) is sequentially reduced in normal-nonpolypoid-polypoid-cancer and in colon cancer cell lines, indicating that *Tmigd1* might be involved in intestinal cell differentiation [27]. *Tmigd1* is downregulated in pseudomyxoma peritonei compared with normal colonic mucosa [28].

*9030624O13Rik* is also known as *Slc9a3* (solute carrier family 9, member 3) or *Nhe3* (sodium-hydrogen exchanger 3). This gene participates in Na^+^/H^+^ exchange in the intestine and the kidney, and impairment of the exchanger deregulates acid-base balance and homeostasis, resulting in diarrhea in the colon [29]. A recent study showed that the decreased level of *Nhe3* is related with inflammatory bowel disease (IBD)-associated diarrhea, suggesting its potential as a therapeutic target [30]. The expression of *Slc9a3* is downregulated in ulcerative colitis patients and its expression is related with the degree of acute inflammation in the colon [31]. *Slc9a3* knockout mice show increased bacterial penetration, inflammation, and spontaneous development of colitis, in addition to reduced colonic microbial diversity [32–34]. Accordingly, we speculated that downregulation of *9030624O13Rik* would be related with the obesity-induced pathophysiological changes in the mouse colon. Furthermore, *Akr1b7* (aldo-keto reductase family 1, member B7) encoding an aldose-reductase is induced by farnesoid X receptor in the small intestine, the colon, and the liver to detoxify bile acids [35], which are known to promote tumor formation in the colon. *Akr1b7* knockout mice show increased adipose tissue and sensitivity to diet-induced obesity, suggesting that *Akr1b7* possesses an anti-adipogenic effect [36]. Thus, downregulation of *Akr1b7* might be associated with adipose tissue accumulation, leading to the development of obesity-induced colonic diseases.

Accumulating evidence from clinical and animal studies suggests that obesity-associated molecules including leptin, insulin, IGF-1, and adiponectin influence the development of colonic diseases. Leptin increases the pro-inflammatory immune responses and promotes colon carcinogenesis by activating the PI3K/Akt signaling pathway [8, 10]. Serum leptin levels in patients with ulcerative colitis are higher compared with those in healthy controls [37]. Leptin expression also increases in the colon tissues of patients with ulcerative colitis and Crohn’s disease [38]. Leptin activates NF-κB involved in the pro-inflammatory stimuli *in vitro*, which are associated with IBD pathogenesis [38]. Indeed, leptin administration induces colonic inflammation *in vivo* [38]. A recent study reported that serum insulin levels are also higher in patients with IBD than those in control individuals, and they are even higher in patients with active IBD than those in patients with inactive IBD [39]. In another study, about 60% of the patients with IBD showed increased insulin levels, whereas serum glucose levels were normal [40]. In addition, IGF-1 is upregulated in colorectal tumor tissues, whereas adiponectin is known to prevent colon tumor growth [11, 12]. However, underlying molecular interactions between these molecules and inflammatory events are not well understood. Therefore, it is important to identify candidate genes as potential early biomarkers for the prevention, early detection, and treatment of these diseases.

We further identified serum phenotype-associated DEGs in the colon with an inverse relationship between the ND and HFD groups in order to investigate the potential early biomarker candidates of obesity-associated colonic diseases. We found that several serum leptin-associated genes including *Peli3*, *Creb1*, and *Enpp2* and serum insulin-associated genes such as *Centg1* are associated with obesity and colonic diseases. *Peli3* encodes Pellino E3 ubiquitin protein ligase family member 3, one of the Pellino proteins that interact with Toll-like receptor (TLR)/interleukin-1 (IL-1) receptor signaling [41]. TLR signaling is known to promote obesity-induced resistance to insulin and leptin [42, 43], suggesting that upregulation of *Peli3* in response to HFD is implicated in the increased serum concentrations of insulin and leptin observed in the present study. Given that an inverse relationship existed between higher serum leptin concentration and the expression of *Peli3* based on the diet types in this study, it seems that circulating leptin concentrations would influence *Peli3* expression, thus highlighting the importance of maintaining normal leptin levels. When *Peli3* was suppressed in murine macrophages, the expression of lipopolysaccharide (LPS)-induced inflammatory cytokines such as TNF-α and IL-1β was decreased [44]. Taken together, these results indicate that *Peli3* could be a potential early biomarker of obesity-induced colonic diseases.

cAMP responsive element binding protein 1 (CREB1) encoded by the *Creb1* gene is a transcription factor that regulates the expression of genes involved in food intake and energy expenditure [45, 46]. The hypothalamic mRNA level of *Creb1* is lower in *ob/ob* mice compared with that in lean mice [46]. An *in vivo* study reported that *Creb1* deficiency in the hypothalamus induces obesity by decreasing energy expenditure [47]. *Creb1* knockout mice fed with a HFD show increase in body weight, fat weight, and serum leptin levels as well as impaired brown adipose tissue activation compared with those on a chow diet [47]. Furthermore, *Enpp2* encodes ectonucleotide pyrophosphatase/phosphodiesterase 2 (Enpp2), also known as autotaxin, which is involved in the conversion of lysophospholipids to lysophosphatidic acid (LPA) [48, 49]. LPA suppresses bacterial endotoxin-induced pro-inflammatory responses, suggesting that it has an anti-inflammatory activity [50]. The function of autotaxin has not been well established. However, a recent *in vitro* study suggested that autotaxin overexpression reduces LPS-induced cytokines including TNF-α and IL-6 [51]. It has also been reported that ENPP2 levels in the serum and in subcutaneous fat decrease in obese humans, indicating that it might be an important factor related with BMI [52]. Collectively, downregulation of *Creb1* and *Enpp2* and an inverse relationship between serum leptin concentration and the expression of these genes by diet type, suggest that different concentration ranges of circulating leptin would affect the regulation of *Creb1* and *Enpp2* differently, presumably explaining the relationship between obesity-induced increases in serum leptin and the pathophysiological events in the colon.

*Centg1* encodes phosphoinositide-3-kinase enhancer (PIKE) protein and GTPase family. PIKE is known as a proto-oncogene in tumor progression due to its anti-apoptotic activity [53]. PIKE is upregulated in a number of human cancer tissues including the colon and enhances cell transformation by Akt activation [54]. Increased levels of *Centg1* are found in various tumors, which lead to tumor invasion [55]. In addition, PIKE is involved in obesity development since it plays a role in insulin-suppressed AMPK activation [56]. PIKE expression increases significantly in the adipose tissue and muscles of obese mice, whereas *PIKE* knockout mice are resistant to HFD-induced obesity due to improved insulin sensitivity as a result of increased AMPK activity [56]. Accordingly, upregulation of *Centg1* in HFD and the inverse relationship between serum insulin concentrations and *Centg1* expression by diet type indicate that above normal insulin concentrations would affect the expression of *Centg1*, which is related with an increased risk of obesity-induced colorectal cancer.

Obesity induces chronic inflammation in the multi-organ involving adipose tissue, the liver, and skeletal muscle, leading to dysbiosis in the intestine [57]. In this study, we investigated the association of serum phenotypes with gene expression profile mainly focused on the colonic tissue, which could be a weakness of this study. Although the use of a less stringent threshold due to a small sample size is a limitation of our study, our confirmation of the ‘less statistically significant’ genes through RT-qPCR provides biological validity of selected genes.

In conclusion, we identified several serum phenotype-associated genes that might affect obesity-induced pathophysiological changes in the colon. Genome-wide profiling and the bioinformatics analyses performed in this study provide evidence for early biomarker candidates of obesity-associated colonic diseases. Future studies verifying the function of these potential candidates are needed for the prevention, early detection, and treatment of colonic diseases.

## Supporting information

S1 Table List of differentially expressed genes in the colon tissue of high-fat diet fed C57BL/6J mice. (DOCX)

S2 Table List of differentially expressed and serum leptin-associated genes in the colon tissue of high-fat diet fed C57BL/J mice. (DOCX)

S3 Table List of differentially expressed and serum insulin-associated genes in the colon tissue of high-fat diet fed C57BL/J mice. (DOCX)

S4 Table List of differentially expressed and serum IGF-1-associated genes in the colon tissue of high-fat diet fed C57BL/J mice. (DOCX)

S5 Table List of differentially expressed and serum adiponectin-associated genes in the colon tissue of high-fat diet fed C57BL/J mice. (DOCX)
