# Supplementary material for: Genome-wide analysis identifies colonic genes differentially associated with serum leptin and insulin concentrations in C57BL/6J mice fed a high-fat diet
Source: PLoS One. 2017 Feb 7;12(2):e0171664. doi: 10.1371/journal.pone.0171664 (PMC5295695; doi:10.1371/journal.pone.0171664)
Supplement: S1 Table — (DOCX) [file pone.0171664.s001.docx]

**S1 Table. List of differentially expressed genes in the colon tissue of high-fat diet fed C57BL/6J mice.**

|  | **Accession** | **Symbol** | **Log_2_ fold change** | **P-value** | **FDR** |
| --- | --- | --- | --- | --- | --- |
| **Up-regulation** | XM_001474081.1 | *LOC383196** | 1.0886 | 0.0305 | 0.9998 |
|  | NM_001039562.1 | *Ankrd37* | 0.9776 | 0.0361 | 0.9998 |
|  | NM_009477.1 | *Upp1** | 0.7288 | 0.0306 | 0.9998 |
|  | NM_009264.2 | *Sprr1a* | 0.7181 | 0.0004 | 0.9321 |
|  | XM_135511 | *9030605I04Rik* | 0.7021 | 0.0078 | 0.9998 |
|  | AK083478 | *Slc11a2* | 0.6506 | 0.0340 | 0.9998 |
|  | NM_010431.1 | *Hif1a* | 0.6204 | 0.0041 | 0.9659 |
|  | NM_174865.1 | *Klk15* | 0.6053 | 0.0186 | 0.9998 |
|  | XR_031459.1 | *LOC100045250* | 0.6007 | 0.0447 | 0.9998 |
| **Down-regulation** | XM_982144.1 | *LOC631037* | -1.2916 | 0.0402 | 0.9998 |
|  | NM_013467.3 | *Aldh1a1* | -1.2109 | 0.0060 | 0.9998 |
|  | NM_021456.2 | *Ces1* | -1.1910 | 0.0105 | 0.9998 |
|  | NM_010762.4 | *Mal** | -0.9453 | 0.0375 | 0.9998 |
|  | NM_008926.3 | *Prkg2* | -0.9098 | 0.0451 | 0.9998 |
|  | NM_033603.2 | *Amn* | -0.8076 | 0.0389 | 0.9998 |
|  | NM_025655.2 | *Tmigd1* | -0.7966 | 0.0423 | 0.9998 |
|  | NM_173404.2 | *Bmp3* | -0.7821 | 0.0337 | 0.9998 |
|  | NM_026183.2 | *1300013J15Rik* | -0.7698 | 0.0370 | 0.9998 |
|  | NM_019823.3 | *Cyp2d22* | -0.7684 | 0.0027 | 0.9321 |
|  | XM_127434 | *9030624O13Rik* | -0.7392 | 0.0491 | 0.9998 |
|  | NM_009731.1 | *Akr1b7* | -0.7330 | 0.0013 | 0.9321 |
|  | NM_175535.3 | *Arhgap20** | -0.6796 | 0.0287 | 0.9998 |
|  | NM_026085.2 | *3110049J23Rik* | -0.6182 | 0.0249 | 0.9998 |
|  | NM_019738.1 | *Nupr1* | -0.6095 | 0.0021 | 0.9321 |
|  | NM_024264.3 | *Cyp27a1* | -0.5973 | 0.0467 | 0.9998 |

Differentially expressed genes showed a t-test P-value < 0.05 and a log_2_ fold change of ≥ 0.5849 (fold change ≥ 1.5) or ≤ -0.5849 (fold change ≤ 0.66) based on the high-fat diet vs. normal diet comparison; A less stringent threshold was applied for such screening due to sample size (n=3 for the ND group; n=6 for the HFD group). However, these statistically significant candidate genes were later technically validated through real-time quantitative polymerase chain reaction to resolve false-positive issues; * A given gene is represented in the microarray set with multiple identifiers; FDR, false discovery rate using a Benjamini and Hochberg multiple testing correction.
