# Supplementary material for: Genome-wide analysis identifies colonic genes differentially associated with serum leptin and insulin concentrations in C57BL/6J mice fed a high-fat diet
Source: PLoS One. 2017 Feb 7;12(2):e0171664. doi: 10.1371/journal.pone.0171664 (PMC5295695; doi:10.1371/journal.pone.0171664)
Supplement: S2 Table — (DOCX) [file pone.0171664.s002.docx]

**S2 Table. List of differentially expressed and serum leptin-associated genes in the colon tissue of high-fat diet fed C57BL/J mice.**

1. Inverse relationships between two dietary groups

| Accession | Symbol | Definition | P-value (interaction; M_PAG1_) | FDR  (interaction) | P-value (t-test; M_DEG_) | | FDR  (t-test) | |
| --- | --- | --- | --- | --- | --- | --- | --- | --- |
| NM_013675.3 | *Spnb1* | Mus musculus spectrin beta 1 (Spnb1), mRNA. | 0.0016 | 0.9837 | 0.0350 | 0.9998 | |  |
| NM_133828.2 | *Creb1* | Mus musculus cAMP responsive element binding protein 1 (Creb1), transcript variant A, mRNA. | 0.0032 | 0.9837 | 0.0372 | 0.9998 | |  |
| NM_080728.2 | *Myh7* | Mus musculus myosin, heavy polypeptide 7, cardiac muscle, beta (Myh7), mRNA. | 0.0096 | 0.9837 | 0.0431 | 0.9998 | |  |
| NM_146764 | *Olfr1408* |  | 0.0127 | 0.9837 | 0.0280 | 0.9998 | |  |
| AK038940 | *Enpp2* |  | 0.0197 | 0.9837 | 0.0209 | 0.9998 | |  |
| AK053156.1 | *scl0002720.1_68* | ILMN_186352 | 0.0257 | 0.9837 | 0.0487 | 0.9998 | |  |
| NM_026312.4 | *2610029G23Rik* | Mus musculus RIKEN cDNA 2610029G23 gene (2610029G23Rik), mRNA. | 0.0264 | 0.9837 | 0.0029 | 0.9321 | |  |
| NM_027168.2 | *Hddc2* | Mus musculus HD domain containing 2 (Hddc2), mRNA. | 0.0265 | 0.9837 | 0.0156 | 0.9998 | |  |
| XM_357535.1 | *LOC384276* | ILMN_200423 | 0.0269 | 0.9839 | 0.0030 | 0.9383 | |  |
| XM_001476869.1 | *E530015N03Rik* | PREDICTED: Mus musculus RIKEN cDNA E530015N03 gene (E530015N03Rik), mRNA. | 0.0273 | 0.9837 | 0.0056 | 0.9998 | |  |
| XR_031436.1 | *LOC676420* | PREDICTED: Mus musculus similar to ceramide kinases (LOC676420), misc RNA. | 0.0289 | 0.9837 | 0.0238 | 0.9998 | |  |
| NM_019789.2 | *Kcnip3* | Mus musculus Kv channel interacting protein 3, calsenilin (Kcnip3), mRNA. | 0.0296 | 0.9837 | 0.0296 | 0.9998 | |  |
| NM_172835.2 | *Peli3* | Mus musculus pellino 3 (Peli3), mRNA. | 0.0312 | 0.9837 | 0.0153 | 0.9998 | |  |
| AK048773 | *Kcnma1* |  | 0.0385 | 0.9837 | 0.0106 | 0.9998 | |  |
| NM_025616.3 | *Timm50* | Mus musculus translocase of inner mitochondrial membrane 50 homolog (yeast) (Timm50), nuclear gene encoding mitochondrial protein, mRNA. | 0.0389 | 0.9837 | 0.0318 | 0.9998 | |  |
| XM_907275.3 | *EG632964* | PREDICTED: Mus musculus predicted gene, EG632964 (EG632964), mRNA. | 0.0413 | 0.9837 | 0.0063 | 0.9998 | |  |
| NM_153054.2 | *Slc18a1* | Mus musculus solute carrier family 18 (vesicular monoamine), member 1 (Slc18a1), mRNA. | 0.0482 | 0.9837 | 0.0007 | 0.9321 | |  |

1. Association between gene expression and serum leptin concentration show no difference among the two dietary groups

| Accession | Symbol | Definition | P-value (interaction; M_PAG1_) | P-value (t-test; M_DEG_) | P-value (no interaction; M_PAG2_) | FDR  (no interaction) |
| --- | --- | --- | --- | --- | --- | --- |
| XM_207109.3 | *LOC280096* | ILMN_198060 | 0.7849 | 0.0283 | 0.0008 | 0.9976 |
| NM_177744.3 | *9030421J09Rik* | Mus musculus RIKEN cDNA 9030421J09 gene (9030421J09Rik), mRNA. | 0.7142 | 0.0403 | 0.0009 | 0.9976 |
| NM_020000.2 | *Med8* | Mus musculus mediator of RNA polymerase II transcription, subunit 8 homolog (yeast) (Med8), transcript variant 1, mRNA. | 0.1737 | 0.0384 | 0.0012 | 0.9976 |
| NR_003269.1 | *Obox2* | Mus musculus oocyte specific homeobox 2 (Obox2), non-coding RNA. | 0.3329 | 0.0278 | 0.0014 | 0.9976 |
| AK006776 | *1700052N19Rik* | ILMN_202189 | 0.2279 | 0.0100 | 0.0016 | 0.9976 |
| AK034046.1 | *scl0003131.1_3* | ILMN_184796 | 0.2891 | 0.0477 | 0.0025 | 0.9976 |
| NM_026332.3 | *Dnajc19* | Mus musculus DnaJ (Hsp40) homolog, subfamily C, member 19 (Dnajc19), transcript variant 1, mRNA. | 0.7297 | 0.0269 | 0.0025 | 0.9976 |
| AK042365 | *A630085E16Rik* | ILMN_205575 | 0.9398 | 0.0122 | 0.0029 | 0.9976 |
| NM_010500.1 | *Ier5* | Mus musculus immediate early response 5 (Ier5), mRNA. | 0.3922 | 0.0310 | 0.0033 | 0.9976 |
| XM_357061.1 | *LOC383443* | ILMN_200680 | 0.4565 | 0.0007 | 0.0035 | 0.9976 |
| AK014396 | *3632411M23Rik* | ILMN_202249 | 0.9491 | 0.0171 | 0.0037 | 0.9976 |
| NM_175490.3 | *Gpr75* | Mus musculus G protein-coupled receptor 75 (Gpr75), mRNA. | 0.2969 | 0.0342 | 0.0039 | 0.9976 |
| NM_001001295.1 | *Dis3l* | Mus musculus DIS3 mitotic control homolog (S. cerevisiae)-like (Dis3l), transcript variant 1, mRNA. | 0.8743 | 0.0234 | 0.0042 | 0.9976 |
| NM_008812.1 | *Padi2* | Mus musculus peptidyl arginine deiminase, type II (Padi2), mRNA. | 0.8950 | 0.0147 | 0.0046 | 0.9976 |
| NM_146885.1 | *Olfr1294* | Mus musculus olfactory receptor 1294 (Olfr1294), mRNA. | 0.9211 | 0.0267 | 0.0066 | 0.9976 |
| NM_029331.2 | *1700019G17Rik* | Mus musculus RIKEN cDNA 1700019G17 gene (1700019G17Rik), mRNA. | 0.4207 | 0.0447 | 0.0067 | 0.9976 |
| BC004695 | *Zfp64* |  | 0.8455 | 0.0009 | 0.0069 | 0.9976 |
| NM_001001983.1 | *Pi4ka* | Mus musculus phosphatidylinositol 4-kinase, catalytic, alpha polypeptide (Pi4ka), mRNA. | 0.8219 | 0.0264 | 0.0069 | 0.9976 |
| NM_027462.4 | *Wars2* | Mus musculus tryptophanyl tRNA synthetase 2 (mitochondrial) (Wars2), nuclear gene encoding mitochondrial protein, mRNA. | 0.6339 | 0.0378 | 0.0069 | 0.9976 |
| NM_145391.1 | *Tapbpl* | Mus musculus TAP binding protein-like (Tapbpl), mRNA. | 0.3334 | 0.0244 | 0.0072 | 0.9976 |
| XM_001477492.1 | *LOC100047126* | PREDICTED: Mus musculus similar to Sctr protein, transcript variant 1 (LOC100047126), mRNA. | 0.6007 | 0.0223 | 0.0074 | 0.9976 |
| AK054364 | *E330019I03Rik* | ILMN_205263 | 0.9291 | 0.0402 | 0.0076 | 0.9976 |
| XM_147733.1 | *1700026N04Rik* | ILMN_196415 | 0.6963 | 0.0363 | 0.0078 | 0.9976 |
| AK037724 | *A130041O12Rik* | ILMN_203762 | 0.7483 | 0.0283 | 0.0080 | 0.9976 |
| NM_010763.1 | *Man1a2* | Mus musculus mannosidase, alpha, class 1A, member 2 (Man1a2), mRNA. | 0.5421 | 0.0274 | 0.0080 | 0.9976 |
| XM_919195.3 | *4921524L21Rik* | PREDICTED: Mus musculus RIKEN cDNA 4921524L21 gene, transcript variant 4 (4921524L21Rik), mRNA. | 0.6631 | 0.0089 | 0.0083 | 0.9976 |
| NM_001004363 | *B230104P22Rik* | ILMN_192574 | 0.5037 | 0.0027 | 0.0090 | 0.9976 |
| NM_138656.1 | *Mvd* | Mus musculus mevalonate (diphospho) decarboxylase (Mvd), mRNA. | 0.8029 | 0.0188 | 0.0091 | 0.9976 |
| NM_147097.1 | *Olfr628* | Mus musculus olfactory receptor 628 (Olfr628), mRNA. | 0.6635 | 0.0495 | 0.0097 | 0.9976 |
| NM_198031.1 | *Tubgcp3* | Mus musculus tubulin, gamma complex associated protein 3 (Tubgcp3), mRNA. | 0.2737 | 0.0028 | 0.0101 | 0.9976 |
| NM_175751.3 | *Zfp608* | Mus musculus zinc finger protein 608 (Zfp608), mRNA. XM_001000874 XM_001000888 XM_001000902 XM_001000914 XM_993937 XM_993952 XM_993993 XM_994038 XM_994057 XM_994076 | 0.7675 | 0.0325 | 0.0101 | 0.9976 |
| NM_146795.1 | *Olfr812* | Mus musculus olfactory receptor 812 (Olfr812), mRNA. | 0.6309 | 0.0450 | 0.0101 | 0.9976 |
| AK051805 | *D130099D04Rik* | ILMN_204932 | 0.5328 | 0.0488 | 0.0106 | 0.9976 |
| AK037506 | *A130022G24Rik* | ILMN_204838 | 0.6695 | 0.0470 | 0.0109 | 0.9976 |
|  | *MJ-3000-112_1692* | ILMN_187329 | 0.5392 | 0.0155 | 0.0111 | 0.9976 |
| NM_177608.3 | *3110001I20Rik* | Mus musculus RIKEN cDNA 3110001I20 gene (3110001I20Rik), mRNA. | 0.5898 | 0.0049 | 0.0116 | 0.9976 |
| NM_021351.1 | *Cryba4* | Mus musculus crystallin, beta A4 (Cryba4), mRNA. | 0.8954 | 0.0151 | 0.0122 | 0.9976 |
| NM_023042.2 | *Recql* | Mus musculus RecQ protein-like (Recql), mRNA. | 0.3144 | 0.0033 | 0.0125 | 0.9976 |
| NM_027978.1 | *Coq2* | Mus musculus coenzyme Q2 homolog, prenyltransferase (yeast) (Coq2), mRNA. | 0.1191 | 0.0109 | 0.0132 | 0.9976 |
| NM_001033540.2 | *EG330503* | Mus musculus predicted gene, EG330503 (EG330503), mRNA. | 0.3312 | 0.0115 | 0.0135 | 0.9976 |
| XM_356568.1 | *LOC382557* | ILMN_201369 | 0.5796 | 0.0187 | 0.0137 | 0.9976 |
| NM_013521.2 | *Fpr1* | Mus musculus formyl peptide receptor 1 (Fpr1), mRNA. | 0.4716 | 0.0187 | 0.0142 | 0.9976 |
| NM_203660.1 | *EG368203* | Mus musculus predicted gene, EG368203 (EG368203), mRNA. | 0.7038 | 0.0356 | 0.0146 | 0.9976 |
| NM_011776.1 | *Zp3* | Mus musculus zona pellucida glycoprotein 3 (Zp3), mRNA. | 0.6625 | 0.0492 | 0.0155 | 0.9976 |
| NM_146886.1 | *Olfr1298* | Mus musculus olfactory receptor 1298 (Olfr1298), mRNA. | 0.9616 | 0.0108 | 0.0155 | 0.9976 |
| NM_023598 | *Arid5b* |  | 0.2458 | 0.0078 | 0.0170 | 0.9976 |
| XM_001474585.1 | *EG434758* | PREDICTED: Mus musculus predicted gene, EG434758 (EG434758), mRNA. | 0.7349 | 0.0036 | 0.0178 | 0.9976 |
| NM_146597.1 | *Olfr702* | Mus musculus olfactory receptor 702 (Olfr702), mRNA. | 0.6285 | 0.0421 | 0.0180 | 0.9976 |
| XM_486217.5 | *EG434401* | PREDICTED: Mus musculus predicted gene, EG434401 (EG434401), mRNA. | 0.3658 | 0.0461 | 0.0190 | 0.9976 |
| NM_019488.4 | *Slc2a8* | Mus musculus solute carrier family 2, (facilitated glucose transporter), member 8 (Slc2a8), mRNA. | 0.3353 | 0.0321 | 0.0193 | 0.9976 |
| NM_001024205.1 | *Nufip2* | Mus musculus nuclear fragile X mental retardation protein interacting protein 2 (Nufip2), mRNA. | 0.4596 | 0.0264 | 0.0194 | 0.9976 |
| XM_136701.2 | *LOC226955* | ILMN_197128 | 0.2905 | 0.0215 | 0.0196 | 0.9976 |
| NM_022326.2 | *Ctsm* | Mus musculus cathepsin M (Ctsm), mRNA. | 0.5523 | 0.0056 | 0.0200 | 0.9976 |
| NM_026626.2 | *Efcab2* | Mus musculus EF-hand calcium binding domain 2 (Efcab2), mRNA. | 0.7416 | 0.0147 | 0.0201 | 0.9976 |
| NM_001048057.1 | *Rpl38* | Mus musculus ribosomal protein L38 (Rpl38), transcript variant 1, mRNA. | 0.7338 | 0.0465 | 0.0202 | 0.9976 |
| XR_004731.1 | *LOC675098* | PREDICTED: Mus musculus similar to jumonji domain containing 2D (LOC675098), mRNA. | 0.1456 | 0.0104 | 0.0207 | 0.9976 |
| NM_026604.3 | *Fam135a* | Mus musculus family with sequence similarity 135, member A (Fam135a), mRNA. | 0.6201 | 0.0113 | 0.0207 | 0.9976 |
| NM_007628.1 | *Ccna1* | Mus musculus cyclin A1 (Ccna1), mRNA. | 0.9460 | 0.0027 | 0.0208 | 0.9976 |
| NM_011536 | *Tbx4* |  | 0.1940 | 0.0481 | 0.0213 | 0.9976 |
| NM_021510.1 | *Hnrph1* | ILMN_217576 | 0.0903 | 0.0401 | 0.0219 | 0.9976 |
| NM_134000.3 | *Traf3ip2* | Mus musculus Traf3 interacting protein 2 (Traf3ip2), mRNA. | 0.9840 | 0.0323 | 0.0229 | 0.9976 |
| NM_053176.1 | *Hrg* | Mus musculus histidine-rich glycoprotein (Hrg), mRNA. | 0.5333 | 0.0076 | 0.0233 | 0.9976 |
| AK019878 | *5031425D22Rik* | ILMN_202773 | 0.8506 | 0.0092 | 0.0238 | 0.9976 |
| AK041682 | *A630029M15Rik* | ILMN_203868 | 0.8799 | 0.0202 | 0.0241 | 0.9976 |
| XM_140032.2 | *LOC210143* | ILMN_198180 | 0.4185 | 0.0343 | 0.0245 | 0.9976 |
| XM_488675 | *AI850995* | ILMN_216535 | 0.3022 | 0.0013 | 0.0257 | 0.9976 |
| NM_001025305.1 | *Tcfap2b* | Mus musculus transcription factor AP-2 beta (Tcfap2b), transcript variant 2, mRNA. | 0.7823 | 0.0441 | 0.0271 | 0.9976 |
| NM_028964.3 | *Snx29* | Mus musculus sorting nexin 29 (Snx29), mRNA. | 0.9791 | 0.0112 | 0.0271 | 0.9976 |
| AK086629 | *D930042N17Rik* | ILMN_206534 | 0.7312 | 0.0003 | 0.0282 | 0.9976 |
|  | *MJ-1000-56_266* | ILMN_194618 | 0.3874 | 0.0221 | 0.0283 | 0.9976 |
| XM_359179 | *1700008H02Rik* | ILMN_188746 | 0.4269 | 0.0090 | 0.0286 | 0.9976 |
| NM_001081414.1 | *Grm5* | Mus musculus glutamate receptor, metabotropic 5 (Grm5), mRNA. | 0.7476 | 0.0141 | 0.0289 | 0.9976 |
| NM_145537.1 | *Edem2* | Mus musculus ER degradation enhancer, mannosidase alpha-like 2 (Edem2), mRNA. | 0.9435 | 0.0187 | 0.0290 | 0.9976 |
| AK017854 | *5730564E11Rik* | ILMN_201847 | 0.6846 | 0.0341 | 0.0295 | 0.9976 |
| NM_010844.1 | *Muc5ac* | Mus musculus mucin 5, subtypes A and C, tracheobronchial/gastric (Muc5ac), mRNA. | 0.9496 | 0.0262 | 0.0297 | 0.9976 |
| NM_016677.3 | *Hpcal1* | Mus musculus hippocalcin-like 1 (Hpcal1), mRNA. | 0.7067 | 0.0343 | 0.0307 | 0.9976 |
| NM_178072.2 | *Glcci1* | Mus musculus glucocorticoid induced transcript 1 (Glcci1), transcript variant 2, mRNA. | 0.3009 | 0.0024 | 0.0308 | 0.9976 |
| NM_172627.3 | *Pggt1b* | Mus musculus protein geranylgeranyltransferase type I, beta subunit (Pggt1b), mRNA. | 0.2822 | 0.0470 | 0.0310 | 0.9976 |
| XM_130322 | *Ttn* |  | 0.7451 | 0.0165 | 0.0315 | 0.9976 |
| NM_001037941.1 | *Dnajb6* | Mus musculus DnaJ (Hsp40) homolog, subfamily B, member 6 (Dnajb6), transcript variant 2, mRNA. | 0.7731 | 0.0083 | 0.0321 | 0.9976 |
| NM_173769.3 | *Zfp641* | Mus musculus zinc finger protein 641 (Zfp641), mRNA. | 0.9832 | 0.0257 | 0.0322 | 0.9976 |
| NM_028623.2 | *Cst6* | Mus musculus cystatin E/M (Cst6), mRNA. | 0.3086 | 0.0156 | 0.0327 | 0.9976 |
| NM_001033211.1 | *AU022751* | Mus musculus expressed sequence AU022751 (AU022751), mRNA. | 0.1589 | 0.0099 | 0.0331 | 0.9976 |
| NM_001013607.1 | *Vmo1* | Mus musculus vitelline membrane outer layer 1 homolog (chicken) (Vmo1), mRNA. | 0.8303 | 0.0240 | 0.0332 | 0.9976 |
| AK008930 | *Anxa10* |  | 0.9972 | 0.0383 | 0.0335 | 0.9976 |
| NM_027544.1 | *Ggnbp1* | Mus musculus gametogenetin binding protein 1 (Ggnbp1), mRNA. | 0.8981 | 0.0166 | 0.0336 | 0.9976 |
| NM_175229.3 | *Srrm2* | Mus musculus serine/arginine repetitive matrix 2 (Srrm2), mRNA. | 0.9776 | 0.0209 | 0.0336 | 0.9976 |
| NM_001081375.1 | *Cnfn* | Mus musculus cornifelin (Cnfn), transcript variant 2, mRNA. | 0.6509 | 0.0472 | 0.0338 | 0.9976 |
| AK078287 | *6430573D20Rik* | ILMN_206996 | 0.9474 | 0.0492 | 0.0338 | 0.9976 |
| XM_142623.3 | *LOC245147* | ILMN_199311 | 0.9454 | 0.0044 | 0.0342 | 0.9976 |
| XM_284369.2 | *9330101J02Rik* | ILMN_223060 | 0.7835 | 0.0492 | 0.0346 | 0.9976 |
| XM_194577.3 | *LOC270491* | ILMN_197807 | 0.6550 | 0.0406 | 0.0355 | 0.9976 |
| XM_001478815.1 | *Omt2a* | PREDICTED: Mus musculus oocyte maturation, alpha, transcript variant 1 (Omt2a), mRNA. | 0.5507 | 0.0404 | 0.0366 | 0.9976 |
| XM_001477781.1 | *LOC100042550* | PREDICTED: Mus musculus similar to putative, transcript variant 1 (LOC100042550), mRNA. | 0.8725 | 0.0344 | 0.0393 | 0.9976 |
| XM_147660.1 | *C130076O07Rik* | ILMN_221761 | 0.9841 | 0.0433 | 0.0406 | 0.9976 |
|  | *MJ-5000-155_2934* | ILMN_194643 | 0.9689 | 0.0105 | 0.0407 | 0.9976 |
| XM_132218.3 | *2310002F18Rik* | ILMN_219267 | 0.9774 | 0.0395 | 0.0416 | 0.9976 |
| NM_053245 | *Aipl1* |  | 0.9542 | 0.0429 | 0.0420 | 0.9976 |
| NM_146151.3 | *Tesk2* | Mus musculus testis-specific kinase 2 (Tesk2), mRNA. | 0.8790 | 0.0493 | 0.0420 | 0.9976 |
| NM_028677.3 | *Ppih* | Mus musculus peptidyl prolyl isomerase H (Ppih), mRNA. | 0.7059 | 0.0014 | 0.0423 | 0.9976 |
| NM_173405.2 | *Amz1* | Mus musculus archaelysin family metallopeptidase 1 (Amz1), mRNA. | 0.5488 | 0.0428 | 0.0424 | 0.9976 |
| NM_146815.1 | *Olfr926* | Mus musculus olfactory receptor 926 (Olfr926), mRNA. | 0.4622 | 0.0166 | 0.0424 | 0.9976 |
| NM_207271.1 | *Tdpoz3* | Mus musculus TD and POZ domain containing 3 (Tdpoz3), mRNA. | 0.9165 | 0.0406 | 0.0433 | 0.9976 |
| NM_007862.2 | *Dlg1* | Mus musculus discs, large homolog 1 (Drosophila) (Dlg1), mRNA. | 0.4275 | 0.0019 | 0.0439 | 0.9976 |
| AK048698 | *Csmd1* |  | 0.7966 | 0.0373 | 0.0439 | 0.9976 |
| NM_023750.2 | *Zfp84* | Mus musculus zinc finger protein 84 (Zfp84), mRNA. | 0.8811 | 0.0079 | 0.0444 | 0.9976 |
| NM_029440.3 | *4930434E21Rik* | Mus musculus RIKEN cDNA 4930434E21 gene (4930434E21Rik), mRNA. | 0.3042 | 0.0121 | 0.0446 | 0.9976 |
|  | *IGKV2-112_J00562_Ig_kappa_variable_2-112_55* | ILMN_185204 | 0.9567 | 0.0028 | 0.0459 | 0.9976 |
| XM_357035.1 | *LOC383407* | ILMN_200624 | 0.3906 | 0.0384 | 0.0461 | 0.9976 |
| NM_001042411.1 | *Lepre1* | Mus musculus leprecan 1 (Lepre1), transcript variant 3, mRNA. | 0.9102 | 0.0483 | 0.0467 | 0.9976 |
| NM_011899.3 | *Srp54* | Mus musculus signal recognition particle 54 (Srp54), mRNA. | 0.7012 | 0.0193 | 0.0467 | 0.9976 |
| NM_010665 | *Krt1-2* | ILMN_224273 | 0.0831 | 0.0198 | 0.0475 | 0.9976 |
| NM_001037921.1 | *EG624367* | Mus musculus predicted gene, EG624367 (EG624367), mRNA. | 0.9185 | 0.0244 | 0.0482 | 0.9976 |
| NM_027868.2 | *Slc41a3* | Mus musculus solute carrier family 41, member 3 (Slc41a3), transcript variant 1, mRNA. | 0.6071 | 0.0152 | 0.0488 | 0.9976 |
| AK087173 | *Gfpt1* |  | 0.9806 | 0.0480 | 0.0493 | 0.9976 |
| XM_358724.1 | *LOC382264* | ILMN_200945 | 0.6119 | 0.0232 | 0.0493 | 0.9976 |
| AK084982 | *D430021H21Rik* | ILMN_206934 | 0.3066 | 0.0323 | 0.0493 | 0.9976 |
| NM_001033263.1 | *Centg1* | Mus musculus centaurin, gamma 1 (Centg1), mRNA. | 0.1733 | 0.0021 | 0.0497 | 0.9976 |

FDR, false discovery rate using a Benjamini and Hochberg multiple testing correction.
