# Supplementary material for: Genome-wide analysis identifies colonic genes differentially associated with serum leptin and insulin concentrations in C57BL/6J mice fed a high-fat diet
Source: PLoS One. 2017 Feb 7;12(2):e0171664. doi: 10.1371/journal.pone.0171664 (PMC5295695; doi:10.1371/journal.pone.0171664)
Supplement: S3 Table — (DOCX) [file pone.0171664.s003.docx]

**S3 Table. List of differentially expressed and serum insulin-associated genes in the colon tissue of high-fat diet fed C57BL/J mice.**

1. Inverse relationships between two dietary groups

| Accession | Symbol | Definition | P-value (interaction; MPAG1) | FDR  (interaction) | P-value (t-test; MDEG) | FDR  (t-test) |
| --- | --- | --- | --- | --- | --- | --- |
| NM_001033263.1 | *Centg1* | Mus musculus centaurin, gamma 1 (Centg1), mRNA. | 0.0165 | 0.9999 | 0.0021 | 0.9321 |
| NM_025723.2 | *4921515J06Rik* | Mus musculus RIKEN cDNA 4921515J06 gene (4921515J06Rik), transcript variant 1, mRNA. | 0.0366 | 0.9999 | 0.0277 | 0.9998 |
| NM_013675.3 | *Spnb1* | Mus musculus spectrin beta 1 (Spnb1), mRNA. | 0.0382 | 0.9999 | 0.0350 | 0.9998 |
| AK053156.1 | *scl0002720.1_68* |  | 0.0447 | 0.9999 | 0.0487 | 0.9998 |

1. Association between gene expression and serum insulin concentration show no difference among the two dietary groups

| Accession | Symbol | Definition | P-value (interaction; MPAG1) | P-value (t-test; MDEG) | P-value (no interaction; MPAG2) | FDR  (no interaction) |
| --- | --- | --- | --- | --- | --- | --- |
| NM_175751.3 | *Zfp608* | Mus musculus zinc finger protein 608 (Zfp608), mRNA. | 0.5802 | 0.0325 | 0.0005 | 0.9999 |
| AK054364 | *E330019I03Rik* | ILMN_205263 | 0.6048 | 0.0402 | 0.0022 | 0.9999 |
| NM_053176.1 | *Hrg* | Mus musculus histidine-rich glycoprotein (Hrg), mRNA. | 0.9739 | 0.0076 | 0.0027 | 0.9999 |
| NM_023773.1 | *Mphosph8* | Mus musculus M-phase phosphoprotein 8 (Mphosph8), mRNA. | 0.2572 | 0.0005 | 0.0035 | 0.9999 |
| NM_027978.1 | *Coq2* | Mus musculus coenzyme Q2 homolog, prenyltransferase (yeast) (Coq2), mRNA. | 0.5732 | 0.0109 | 0.0036 | 0.9999 |
| NM_001033540.2 | *EG330503* | Mus musculus predicted gene, EG330503 (EG330503), mRNA. | 0.6933 | 0.0115 | 0.0039 | 0.9999 |
| XM_358141.1 | *LOC385241* | ILMN_201483 | 0.0811 | 0.0246 | 0.0040 | 0.9999 |
| XM_207109.3 | *LOC280096* | ILMN_198060 | 0.2662 | 0.0283 | 0.0041 | 0.9999 |
| NM_172627.3 | *Pggt1b* | Mus musculus protein geranylgeranyltransferase type I, beta subunit (Pggt1b), mRNA. | 0.1850 | 0.0470 | 0.0042 | 0.9999 |
| XM_911841.2 | *Mocs3* | PREDICTED: Mus musculus molybdenum cofactor synthesis 3 (Mocs3), mRNA. | 0.0821 | 0.0011 | 0.0049 | 0.9999 |
| AK014396 | *3632411M23Rik* | ILMN_202249 | 0.5078 | 0.0171 | 0.0052 | 0.9999 |
| NM_145537.1 | *Edem2* | Mus musculus ER degradation enhancer, mannosidase alpha-like 2 (Edem2), mRNA. | 0.4988 | 0.0187 | 0.0056 | 0.9999 |
| NM_172835.2 | *Peli3* | Mus musculus pellino 3 (Peli3), mRNA. | 0.1318 | 0.0153 | 0.0058 | 0.9999 |
| NM_020000.2 | *Med8* | Mus musculus mediator of RNA polymerase II transcription, subunit 8 homolog (yeast) (Med8), transcript variant 1, mRNA. | 0.7544 | 0.0384 | 0.0059 | 0.9999 |
| NM_001081375.1 | *Cnfn* | Mus musculus cornifelin (Cnfn), transcript variant 2, mRNA. | 0.7504 | 0.0472 | 0.0061 | 0.9999 |
| NM_001081336.1 | *Dgkh* | Mus musculus diacylglycerol kinase, eta (Dgkh), mRNA. | 0.1999 | 0.0274 | 0.0068 | 0.9999 |
| NM_009420.1 | *Crisp2* | Mus musculus cysteine-rich secretory protein 2 (Crisp2), mRNA. | 0.8057 | 0.0474 | 0.0070 | 0.9999 |
| NM_007550.3 | *Blm* | Mus musculus Bloom syndrome homolog (human) (Blm), transcript variant 1, mRNA. | 0.9648 | 0.0202 | 0.0070 | 0.9999 |
| AK086629 | *D930042N17Rik* | ILMN_206534 | 0.5709 | 0.0003 | 0.0074 | 0.9999 |
| NM_080728.2 | *Myh7* | Mus musculus myosin, heavy polypeptide 7, cardiac muscle, beta (Myh7), mRNA. | 0.1030 | 0.0431 | 0.0074 | 0.9999 |
| NM_001081236.1 | *2410131K14Rik* | Mus musculus RIKEN cDNA 2410131K14 gene (2410131K14Rik), mRNA. | 0.3194 | 0.0326 | 0.0076 | 0.9999 |
| NM_053245 | *Aipl1* |  | 0.6464 | 0.0429 | 0.0078 | 0.9999 |
| NM_001024205.1 | *Nufip2* | Mus musculus nuclear fragile X mental retardation protein interacting protein 2 (Nufip2), mRNA. | 0.3128 | 0.0264 | 0.0080 | 0.9999 |
| NM_001037941.1 | *Dnajb6* | Mus musculus DnaJ (Hsp40) homolog, subfamily B, member 6 (Dnajb6), transcript variant 2, mRNA. | 0.9977 | 0.0083 | 0.0081 | 0.9999 |
| AK034046.1 | *scl0003131.1_3* | ILMN_184796 | 0.9716 | 0.0477 | 0.0088 | 0.9999 |
| NM_010665 | *Krt1-2* | ILMN_224273 | 0.1686 | 0.0198 | 0.0088 | 0.9999 |
| XM_001474585.1 | *EG434758* | PREDICTED: Mus musculus predicted gene, EG434758 (EG434758), mRNA. | 0.6806 | 0.0036 | 0.0091 | 0.9999 |
| XM_913690.2 | *Gpr39* | PREDICTED: Mus musculus G protein-coupled receptor 39 (Gpr39), mRNA. | 0.2555 | 0.0440 | 0.0094 | 0.9999 |
| NM_026312.4 | *2610029G23Rik* | Mus musculus RIKEN cDNA 2610029G23 gene (2610029G23Rik), mRNA. | 0.1547 | 0.0029 | 0.0096 | 0.9999 |
| NM_001001295.1 | *Dis3l* | Mus musculus DIS3 mitotic control homolog (S. cerevisiae)-like (Dis3l), transcript variant 1, mRNA. | 0.3690 | 0.0234 | 0.0100 | 0.9999 |
| XM_357535.1 | *LOC384276* | ILMN_200423 | 0.3119 | 0.0030 | 0.0101 | 0.9999 |
| XM_001477536.1 | *LOC100042083* | PREDICTED: Mus musculus similar to 2810047C21Rik protein (LOC100042083), mRNA. | 0.7745 | 0.0021 | 0.0104 | 0.9999 |
| NM_001004363 | *B230104P22Rik* | ILMN_192574 | 0.7869 | 0.0027 | 0.0106 | 0.9999 |
| NM_022326.2 | *Ctsm* | Mus musculus cathepsin M (Ctsm), mRNA. | 0.2121 | 0.0056 | 0.0107 | 0.9999 |
| XM_001477976.1 | *LOC100047208* | PREDICTED: Mus musculus similar to trypsinogen 15 (LOC100047208), mRNA. | 0.0509 | 0.0345 | 0.0115 | 0.9999 |
| NM_183417.2 | *Cdk2* | Mus musculus cyclin-dependent kinase 2 (Cdk2), transcript variant 1, mRNA. | 0.1432 | 0.0413 | 0.0116 | 0.9999 |
| NM_001007576.1 | *Gucy2f* | Mus musculus guanylate cyclase 2f (Gucy2f), mRNA. | 0.2931 | 0.0125 | 0.0123 | 0.9999 |
| NM_146885.1 | *Olfr1294* | Mus musculus olfactory receptor 1294 (Olfr1294), mRNA. | 0.9013 | 0.0267 | 0.0124 | 0.9999 |
| NM_178072.2 | *Glcci1* | Mus musculus glucocorticoid induced transcript 1 (Glcci1), transcript variant 2, mRNA. | 0.3562 | 0.0024 | 0.0125 | 0.9999 |
| XM_919195.3 | *4921524L21Rik* | PREDICTED: Mus musculus RIKEN cDNA 4921524L21 gene, transcript variant 4 (4921524L21Rik), mRNA. | 0.6080 | 0.0089 | 0.0131 | 0.9999 |
| NM_053070.1 | *Car7* | Mus musculus carbonic anhydrase 7 (Car7), mRNA. | 0.8770 | 0.0276 | 0.0132 | 0.9999 |
| NM_010500.1 | *Ier5* | Mus musculus immediate early response 5 (Ier5), mRNA. | 0.9369 | 0.0310 | 0.0143 | 0.9999 |
| XM_357035.1 | *LOC383407* | ILMN_200624 | 0.9948 | 0.0384 | 0.0147 | 0.9999 |
| NM_177608.3 | *3110001I20Rik* | Mus musculus RIKEN cDNA 3110001I20 gene (3110001I20Rik), mRNA. | 0.1701 | 0.0049 | 0.0148 | 0.9999 |
| AK041682 | *A630029M15Rik* | ILMN_203868 | 0.5812 | 0.0202 | 0.0149 | 0.9999 |
| NM_010574.2 | *Irx2* | Mus musculus Iroquois related homeobox 2 (Drosophila) (Irx2), mRNA. | 0.4673 | 0.0269 | 0.0151 | 0.9999 |
| NM_029331.2 | *1700019G17Rik* | Mus musculus RIKEN cDNA 1700019G17 gene (1700019G17Rik), mRNA. | 0.2161 | 0.0447 | 0.0155 | 0.9999 |
| AK042365 | *A630085E16Rik* | ILMN_205575 | 0.4906 | 0.0122 | 0.0156 | 0.9999 |
| AK038940 | *Enpp2* |  | 0.1553 | 0.0209 | 0.0157 | 0.9999 |
| NM_145431.1 | *Nle1* | Mus musculus notchless homolog 1 (Drosophila) (Nle1), mRNA. | 0.6923 | 0.0080 | 0.0163 | 0.9999 |
| AK035512 | *9530058K19Rik* | ILMN_203680 | 0.5309 | 0.0166 | 0.0170 | 0.9999 |
| NM_001002896.2 | *Bfsp2* | Mus musculus beaded filament structural protein 2, phakinin (Bfsp2), mRNA. | 0.1421 | 0.0048 | 0.0174 | 0.9999 |
| AK050783 | *4933406L09Rik* | ILMN_205197 | 0.4955 | 0.0129 | 0.0183 | 0.9999 |
| NM_146642.1 | *Olfr1140* | Mus musculus olfactory receptor 1140 (Olfr1140), mRNA. | 0.3106 | 0.0204 | 0.0190 | 0.9999 |
| NM_146358.1 | *Olfr677* | Mus musculus olfactory receptor 677 (Olfr677), mRNA. | 0.0925 | 0.0247 | 0.0190 | 0.9999 |
| XM_140032.2 | *LOC210143* | ILMN_198180 | 0.8580 | 0.0343 | 0.0196 | 0.9999 |
| XM_147733.1 | *1700026N04Rik* | ILMN_196415 | 0.7813 | 0.0363 | 0.0197 | 0.9999 |
| NM_178116.3 | *Camta2* | Mus musculus calmodulin binding transcription activator 2 (Camta2), mRNA. | 0.9079 | 0.0122 | 0.0198 | 0.9999 |
| NM_029440.3 | *4930434E21Rik* | Mus musculus RIKEN cDNA 4930434E21 gene (4930434E21Rik), mRNA. | 0.8065 | 0.0121 | 0.0199 | 0.9999 |
| AK006776 | *1700052N19Rik* | ILMN_202189 | 0.9818 | 0.0100 | 0.0205 | 0.9999 |
| NM_027462.4 | *Wars2* | Mus musculus tryptophanyl tRNA synthetase 2 (mitochondrial) (Wars2), nuclear gene encoding mitochondrial protein, mRNA. | 0.3524 | 0.0378 | 0.0206 | 0.9999 |
| NM_001033434.1 | *Gm884* | Mus musculus gene model 884, (NCBI) (Gm884), mRNA. | 0.1450 | 0.0229 | 0.0210 | 0.9999 |
| XM_142623.3 | *LOC245147* | ILMN_199311 | 0.4033 | 0.0044 | 0.0212 | 0.9999 |
| NM_026626.2 | *Efcab2* | Mus musculus EF-hand calcium binding domain 2 (Efcab2), mRNA. | 0.9156 | 0.0147 | 0.0217 | 0.9999 |
| XM_356568.1 | *LOC382557* | ILMN_201369 | 0.2162 | 0.0187 | 0.0225 | 0.9999 |
| NM_173405.2 | *Amz1* | Mus musculus archaelysin family metallopeptidase 1 (Amz1), mRNA. | 0.1282 | 0.0428 | 0.0231 | 0.9999 |
| XM_357056.1 | *LOC383439* | ILMN_200658 | 0.6967 | 0.0147 | 0.0243 | 0.9999 |
| NM_198031.1 | *Tubgcp3* | Mus musculus tubulin, gamma complex associated protein 3 (Tubgcp3), mRNA. | 0.9579 | 0.0028 | 0.0244 | 0.9999 |
| NM_178041.1 | *Eif5* | Mus musculus eukaryotic translation initiation factor 5 (Eif5), transcript variant 2, mRNA. | 0.5182 | 0.0226 | 0.0244 | 0.9999 |
| AK051805 | *D130099D04Rik* | ILMN_204932 | 0.8826 | 0.0488 | 0.0244 | 0.9999 |
| NM_013521.2 | *Fpr1* | Mus musculus formyl peptide receptor 1 (Fpr1), mRNA. | 0.3331 | 0.0187 | 0.0247 | 0.9999 |
| XM_357061.1 | *LOC383443* | ILMN_200680 | 0.3078 | 0.0007 | 0.0248 | 0.9999 |
| AK017854 | *5730564E11Rik* | ILMN_201847 | 0.9668 | 0.0341 | 0.0253 | 0.9999 |
| NM_013875.2 | *Pde7b* | Mus musculus phosphodiesterase 7B (Pde7b), mRNA. | 0.3622 | 0.0173 | 0.0256 | 0.9999 |
| XM_137040.1 | *EG215895* | PREDICTED: Mus musculus predicted gene, EG215895 (EG215895), mRNA. | 0.8470 | 0.0208 | 0.0259 | 0.9999 |
| NM_138604.2 | *Otud5* | Mus musculus OTU domain containing 5 (Otud5), mRNA. | 0.4380 | 0.0276 | 0.0260 | 0.9999 |
| NM_146477.1 | *Olfr90* | Mus musculus olfactory receptor 90 (Olfr90), mRNA. | 0.6090 | 0.0079 | 0.0260 | 0.9999 |
| NM_146795.1 | *Olfr812* | Mus musculus olfactory receptor 812 (Olfr812), mRNA. | 0.7210 | 0.0450 | 0.0263 | 0.9999 |
| NM_177172 | *A830054O04Rik* | ILMN_194812 | 0.2662 | 0.0180 | 0.0263 | 0.9999 |
| NM_207271.1 | *Tdpoz3* | Mus musculus TD and POZ domain containing 3 (Tdpoz3), mRNA. | 0.8127 | 0.0406 | 0.0271 | 0.9999 |
| AK019878 | *5031425D22Rik* | ILMN_202773 | 0.9333 | 0.0092 | 0.0274 | 0.9999 |
|  | *1700037N05Rik* | ILMN_190174 | 0.8935 | 0.0200 | 0.0280 | 0.9999 |
| XM_001477492.1 | *LOC100047126* | PREDICTED: Mus musculus similar to Sctr protein, transcript variant 1 (LOC100047126), mRNA. | 0.8055 | 0.0223 | 0.0286 | 0.9999 |
| NM_145993.2 | *L3mbtl2* | Mus musculus l(3)mbt-like 2 (Drosophila) (L3mbtl2), mRNA. | 0.1429 | 0.0093 | 0.0289 | 0.9999 |
| NM_026528.3 | *2700060E02Rik* | Mus musculus RIKEN cDNA 2700060E02 gene (2700060E02Rik), mRNA. | 0.3469 | 0.0296 | 0.0289 | 0.9999 |
| NM_146550.1 | *Olfr810* | Mus musculus olfactory receptor 810 (Olfr810), mRNA. | 0.8301 | 0.0089 | 0.0291 | 0.9999 |
| XR_004731.1 | *LOC675098* | PREDICTED: Mus musculus similar to jumonji domain containing 2D (LOC675098), mRNA. | 0.6477 | 0.0104 | 0.0292 | 0.9999 |
| XM_001480092.1 | *LOC100048365* | PREDICTED: Mus musculus similar to keratin associated protein 6-1 (LOC100048365), mRNA. | 0.4070 | 0.0049 | 0.0296 | 0.9999 |
| XM_357073.1 | *LOC383458* | ILMN_200692 | 0.8145 | 0.0127 | 0.0319 | 0.9999 |
| XM_126005.7 | *Pkd1l1* | PREDICTED: Mus musculus polycystic kidney disease 1 like 1 (Pkd1l1), mRNA. | 0.5559 | 0.0247 | 0.0332 | 0.9999 |
| NM_153054.2 | *Slc18a1* | Mus musculus solute carrier family 18 (vesicular monoamine), member 1 (Slc18a1), mRNA. | 0.1814 | 0.0007 | 0.0337 | 0.9999 |
| XM_136701.2 | *LOC226955* | ILMN_197128 | 0.3280 | 0.0215 | 0.0341 | 0.9999 |
| NM_021510.1 | *Hnrph1* | ILMN_217576 | 0.4772 | 0.0401 | 0.0350 | 0.9999 |
| NM_028677.3 | *Ppih* | Mus musculus peptidyl prolyl isomerase H (Ppih), mRNA. | 0.6605 | 0.0014 | 0.0353 | 0.9999 |
| XM_359179 | *1700008H02Rik* | ILMN_188746 | 0.7737 | 0.0090 | 0.0356 | 0.9999 |
| NM_009021.2 | *Rai1* | Mus musculus retinoic acid induced 1 (Rai1), transcript variant 1, mRNA. | 0.3616 | 0.0187 | 0.0358 | 0.9999 |
| AK086955 | *Syn3* |  | 0.3887 | 0.0370 | 0.0367 | 0.9999 |
| NM_147097.1 | *Olfr628* | Mus musculus olfactory receptor 628 (Olfr628), mRNA. | 0.4587 | 0.0495 | 0.0368 | 0.9999 |
| XM_001478815.1 | *Omt2a* | PREDICTED: Mus musculus oocyte maturation, alpha, transcript variant 1 (Omt2a), mRNA. | 0.8732 | 0.0404 | 0.0371 | 0.9999 |
| NM_021351.1 | *Cryba4* | Mus musculus crystallin, beta A4 (Cryba4), mRNA. | 0.6336 | 0.0151 | 0.0376 | 0.9999 |
| NM_020488.1 | *Gabrq* | Mus musculus gamma-aminobutyric acid (GABA-A) receptor, subunit theta (Gabrq), mRNA. | 0.9691 | 0.0051 | 0.0376 | 0.9999 |
| NM_026182.4 | *Mtfr1* | Mus musculus mitochondrial fission regulator 1 (Mtfr1), nuclear gene encoding mitochondrial protein, mRNA. | 0.4544 | 0.0449 | 0.0377 | 0.9999 |
| NM_027868.2 | *Slc41a3* | Mus musculus solute carrier family 41, member 3 (Slc41a3), transcript variant 1, mRNA. | 0.9090 | 0.0152 | 0.0381 | 0.9999 |
| NM_023042.2 | *Recql* | Mus musculus RecQ protein-like (Recql), mRNA. | 0.4540 | 0.0033 | 0.0388 | 0.9999 |
| XM_147660.1 | *C130076O07Rik* | ILMN_221761 | 0.6567 | 0.0433 | 0.0398 | 0.9999 |
| AK054034 | *1700021I09Rik* | ILMN_205420 | 0.8485 | 0.0009 | 0.0404 | 0.9999 |
| NM_025616.3 | *Timm50* | Mus musculus translocase of inner mitochondrial membrane 50 homolog (yeast) (Timm50), nuclear gene encoding mitochondrial protein, mRNA. | 0.2452 | 0.0318 | 0.0416 | 0.9999 |
| NM_008812.1 | *Padi2* | Mus musculus peptidyl arginine deiminase, type II (Padi2), mRNA. | 0.4523 | 0.0147 | 0.0417 | 0.9999 |
| NM_028623.2 | *Cst6* | Mus musculus cystatin E/M (Cst6), mRNA. | 0.5533 | 0.0156 | 0.0428 | 0.9999 |
| AK076385 | *4732481D19Rik* | ILMN_207303 | 0.3327 | 0.0247 | 0.0431 | 0.9999 |
| XM_486217.5 | *EG434401* | PREDICTED: Mus musculus predicted gene, EG434401 (EG434401), mRNA. | 0.1436 | 0.0461 | 0.0435 | 0.9999 |
| NM_009220.1 | *Ssty1* | Mus musculus spermiogenesis specific transcript on the Y 1 (Ssty1), mRNA. | 0.7129 | 0.0207 | 0.0436 | 0.9999 |
| NM_001081005.1 | *1500012F01Rik* | Mus musculus RIKEN cDNA 1500012F01 gene (1500012F01Rik), mRNA. | 0.3801 | 0.0027 | 0.0447 | 0.9999 |
| NM_011400.2 | *Slc2a1* | Mus musculus solute carrier family 2 (facilitated glucose transporter), member 1 (Slc2a1), mRNA. | 0.3458 | 0.0347 | 0.0449 | 0.9999 |
|  | *IGKV2-112_J00562_Ig_kappa_variable_2-112_55* | ILMN_185204 | 0.5753 | 0.0028 | 0.0450 | 0.9999 |
| AK079848 | *A430092C21Rik* | ILMN_203084 | 0.9431 | 0.0128 | 0.0452 | 0.9999 |
|  | *MJ-1000-56_266* | ILMN_194618 | 0.2878 | 0.0221 | 0.0464 | 0.9999 |
| AK004152 | *Znfn1a3* | ILMN_201649 | 0.9205 | 0.0259 | 0.0466 | 0.9999 |
| NM_001033542.1 | *4933429E10Rik* | Mus musculus RIKEN cDNA 4933429E10 gene (4933429E10Rik), mRNA. | 0.3447 | 0.0369 | 0.0467 | 0.9999 |
| XM_140916.2 | *LOC227757* | ILMN_197226 | 0.9773 | 0.0206 | 0.0475 | 0.9999 |
| NM_001033148.2 | *1700029J07Rik* | Mus musculus RIKEN cDNA 1700029J07 gene (1700029J07Rik), mRNA. | 0.7251 | 0.0282 | 0.0477 | 0.9999 |
| NM_146523.1 | *Olfr850* | Mus musculus olfactory receptor 850 (Olfr850), mRNA. | 0.1510 | 0.0475 | 0.0485 | 0.9999 |
| NM_146151.3 | *Tesk2* | Mus musculus testis-specific kinase 2 (Tesk2), mRNA. | 0.3849 | 0.0493 | 0.0487 | 0.9999 |
| NM_138656.1 | *Mvd* | Mus musculus mevalonate (diphospho) decarboxylase (Mvd), mRNA. | 0.4039 | 0.0188 | 0.0499 | 0.9999 |

FDR, false discovery rate using a Benjamini and Hochberg multiple testing correction.
