# Supplementary material for: Genome-wide analysis identifies colonic genes differentially associated with serum leptin and insulin concentrations in C57BL/6J mice fed a high-fat diet
Source: PLoS One. 2017 Feb 7;12(2):e0171664. doi: 10.1371/journal.pone.0171664 (PMC5295695; doi:10.1371/journal.pone.0171664)
Supplement: S4 Table — (DOCX) [file pone.0171664.s004.docx]

**S4 Table. List of differentially expressed and serum IGF-1-associated genes in the colon tissue of high-fat diet fed C57BL/J mice.**

1. Inverse relationships between two dietary groups

| Accession | Symbol | Definition | P-value (interaction; M_PAG1_) | FDR  (interaction) | P-value (t-test; M_DEG_) | FDR  (t-test) |
| --- | --- | --- | --- | --- | --- | --- |
| NM_026312.4 | *2610029G23Rik* | Mus musculus RIKEN cDNA 2610029G23 gene (2610029G23Rik), mRNA. | 0.0454 | 0.7998 | 0.0029 | 0.9321 |
| XM_357535.1 | *LOC384276* | ILMN_200423 | 0.0195 | 0.7998 | 0.0030 | 0.9383 |
| NM_001002896.2 | *Bfsp2* | Mus musculus beaded filament structural protein 2, phakinin (Bfsp2), mRNA. | 0.0195 | 0.7998 | 0.0048 | 0.9978 |
| NM_001081292.1 | *Map3k10* | Mus musculus mitogen-activated protein kinase kinase kinase 10 (Map3k10), mRNA. | 0.0358 | 0.7998 | 0.0070 | 0.9998 |
| NM_145993.2 | *L3mbtl2* | Mus musculus l(3)mbt-like 2 (Drosophila) (L3mbtl2), mRNA. | 0.0275 | 0.7998 | 0.0093 | 0.9998 |
| NM_001033211.1 | *AU022751* | Mus musculus expressed sequence AU022751 (AU022751), mRNA. | 0.0143 | 0.7998 | 0.0099 | 0.9998 |
| XR_004731.1 | *LOC675098* | PREDICTED: Mus musculus similar to jumonji domain containing 2D (LOC675098), mRNA. | 0.0407 | 0.7998 | 0.0104 | 0.9998 |
| AK048773 | *Kcnma1* |  | 0.0437 | 0.7998 | 0.0106 | 0.9998 |
| NM_027978.1 | *Coq2* | Mus musculus coenzyme Q2 homolog, prenyltransferase (yeast) (Coq2), mRNA. | 0.0400 | 0.7998 | 0.0109 | 0.9998 |
| NM_030706.1 | *Trim2* | Mus musculus tripartite motif protein 2 (Trim2), mRNA. XM_984114 XM_984144 XM_984172 XM_984200 XM_984238 XM_984275 XM_984313 | 0.0260 | 0.7998 | 0.0115 | 0.9998 |
| NM_009060.2 | *Rgn* | Mus musculus regucalcin (Rgn), mRNA. | 0.0489 | 0.7998 | 0.0140 | 0.9998 |
| NM_172835.2 | *Peli3* | Mus musculus pellino 3 (Peli3), mRNA. | 0.0000 | 0.1768 | 0.0153 | 0.9998 |
| NM_013875.2 | *Pde7b* | Mus musculus phosphodiesterase 7B (Pde7b), mRNA. | 0.0145 | 0.7998 | 0.0173 | 0.9998 |
| NM_001039710.1 | *Coq10b* | Mus musculus coenzyme Q10 homolog B (S. cerevisiae) (Coq10b), transcript variant 1, mRNA. | 0.0107 | 0.7998 | 0.0237 | 0.9998 |
| AK076385 | *4732481D19Rik* | ILMN_207303 | 0.0262 | 0.7998 | 0.0247 | 0.9998 |
| NM_146358.1 | *Olfr677* | Mus musculus olfactory receptor 677 (Olfr677), mRNA. | 0.0212 | 0.7998 | 0.0247 | 0.9998 |
| NM_032003.1 | *Enpp5* | Mus musculus ectonucleotide pyrophosphatase/phosphodiesterase 5 (Enpp5), mRNA. | 0.0442 | 0.7998 | 0.0260 | 0.9998 |
| NM_010574.2 | *Irx2* | Mus musculus Iroquois related homeobox 2 (Drosophila) (Irx2), mRNA. | 0.0436 | 0.7998 | 0.0269 | 0.9998 |
| NM_001081336.1 | *Dgkh* | Mus musculus diacylglycerol kinase, eta (Dgkh), mRNA. | 0.0261 | 0.7998 | 0.0274 | 0.9998 |
| NM_146764 | *Olfr1408* |  | 0.0260 | 0.7998 | 0.0280 | 0.9998 |
| NM_175347.4 | *Srl* | Mus musculus sarcalumenin (Srl), mRNA. | 0.0397 | 0.7998 | 0.0284 | 0.9998 |
| AK008164 | *2010009J12Rik* | ILMN_202421 | 0.0305 | 0.7998 | 0.0288 | 0.9998 |
| NM_010735.1 | *Lta* | Mus musculus lymphotoxin A (Lta), mRNA. | 0.0312 | 0.7998 | 0.0295 | 0.9998 |
| NM_019789.2 | *Kcnip3* | Mus musculus Kv channel interacting protein 3, calsenilin (Kcnip3), mRNA. | 0.0155 | 0.7998 | 0.0296 | 0.9998 |
| XM_356799.1 | *LOC383012* | ILMN_199786 | 0.0292 | 0.7998 | 0.0324 | 0.9998 |
| NM_001081236.1 | *2410131K14Rik* | Mus musculus RIKEN cDNA 2410131K14 gene (2410131K14Rik), mRNA. | 0.0046 | 0.7998 | 0.0326 | 0.9998 |
| NM_010699.1 | *Ldha* | Mus musculus lactate dehydrogenase A (Ldha), mRNA. | 0.0446 | 0.7998 | 0.0334 | 0.9998 |
| XM_001479095.1 | *LOC100047902* | PREDICTED: Mus musculus similar to RIKEN cDNA 1700022C21 gene (LOC100047902), mRNA. | 0.0427 | 0.7998 | 0.0345 | 0.9998 |
| XM_001477976.1 | *LOC100047208* | PREDICTED: Mus musculus similar to trypsinogen 15 (LOC100047208), mRNA. | 0.0068 | 0.7998 | 0.0345 | 0.9998 |
| NM_011400.2 | *Slc2a1* | Mus musculus solute carrier family 2 (facilitated glucose transporter), member 1 (Slc2a1), mRNA. | 0.0304 | 0.7998 | 0.0347 | 0.9998 |
| NM_009338.3 | *Acat2* | Mus musculus acetyl-Coenzyme A acetyltransferase 2 (Acat2), mRNA. | 0.0288 | 0.7998 | 0.0351 | 0.9998 |
| NM_001033542.1 | *4933429E10Rik* | Mus musculus RIKEN cDNA 4933429E10 gene (4933429E10Rik), mRNA. | 0.0402 | 0.7998 | 0.0369 | 0.9998 |
| AK030051 | *4932418B07Rik* | ILMN_205552 | 0.0430 | 0.7998 | 0.0391 | 0.9998 |
| NM_183417.2 | *Cdk2* | Mus musculus cyclin-dependent kinase 2 (Cdk2), transcript variant 1, mRNA. | 0.0337 | 0.7998 | 0.0413 | 0.9998 |
| XM_913690.2 | *Gpr39* | PREDICTED: Mus musculus G protein-coupled receptor 39 (Gpr39), mRNA. | 0.0391 | 0.7998 | 0.0440 | 0.9998 |
| NM_030721.2 | *Acox3* | Mus musculus acyl-Coenzyme A oxidase 3, pristanoyl (Acox3), mRNA. | 0.0240 | 0.7998 | 0.0445 | 0.9998 |
| NM_026182.4 | *Mtfr1* | Mus musculus mitochondrial fission regulator 1 (Mtfr1), nuclear gene encoding mitochondrial protein, mRNA. | 0.0478 | 0.7998 | 0.0449 | 0.9998 |
| NM_172838.3 | *Slc16a12* | Mus musculus solute carrier family 16 (monocarboxylic acid transporters), member 12 (Slc16a12), mRNA. | 0.0424 | 0.7998 | 0.0455 | 0.9998 |
| NM_028034.2 | *Tdrd12* | Mus musculus tudor domain containing 12 (Tdrd12), transcript variant 2, mRNA. | 0.0326 | 0.7998 | 0.0459 | 0.9998 |
| NM_027976.2 | *Acsl5* | Mus musculus acyl-CoA synthetase long-chain family member 5 (Acsl5), mRNA. | 0.0235 | 0.7998 | 0.0471 | 0.9998 |
| XM_141626.2 | *LOC245350* | ILMN_198536 | 0.0071 | 0.7998 | 0.0472 | 0.9998 |
| NM_007620.2 | *Cbr1* | Mus musculus carbonyl reductase 1 (Cbr1), mRNA. | 0.0490 | 0.7998 | 0.0473 | 0.9998 |
| NM_009863.2 | *Cdc7* | Mus musculus cell division cycle 7 (S. cerevisiae) (Cdc7), mRNA. | 0.0201 | 0.7998 | 0.0478 | 0.9998 |
| NM_028933.1 | *1300010M03Rik* | Mus musculus RIKEN cDNA 1300010M03 gene (1300010M03Rik), transcript variant 2, mRNA. | 0.0188 | 0.7998 | 0.0486 | 0.9998 |

1. Association between gene expression and serum IGF-1 concentration show no difference among the two dietary groups

| Accession | Symbol | Definition | P-value (interaction; M_PAG1_) | P-value (t-test; M_DEG_) | P-value (no interaction; M_PAG2_) | FDR  (no interaction) |
| --- | --- | --- | --- | --- | --- | --- |
| NM_177744.3 | *9030421J09Rik* | Mus musculus RIKEN cDNA 9030421J09 gene (9030421J09Rik), mRNA. | 0.0507 | 0.0403 | 0.0010 | 0.9997 |
| XM_356568.1 | *LOC382557* | ILMN_201369 | 0.3407 | 0.0187 | 0.0024 | 0.9997 |
| NM_177608.3 | *3110001I20Rik* | Mus musculus RIKEN cDNA 3110001I20 gene (3110001I20Rik), mRNA. | 0.3271 | 0.0049 | 0.0028 | 0.9997 |
| NR_003269.1 | *Obox2* | Mus musculus oocyte specific homeobox 2 (Obox2), non-coding RNA. | 0.7668 | 0.0278 | 0.0053 | 0.9997 |
| AK042365 | *A630085E16Rik* | ILMN_205575 | 0.0957 | 0.0122 | 0.0057 | 0.9997 |
| NM_146597.1 | *Olfr702* | Mus musculus olfactory receptor 702 (Olfr702), mRNA. | 0.8513 | 0.0421 | 0.0067 | 0.9997 |
| NM_138656.1 | *Mvd* | Mus musculus mevalonate (diphospho) decarboxylase (Mvd), mRNA. | 0.3567 | 0.0188 | 0.0083 | 0.9997 |
| XM_357061.1 | *LOC383443* | ILMN_200680 | 0.3348 | 0.0007 | 0.0091 | 0.9997 |
| AK034046.1 | *scl0003131.1_3* | ILMN_184796 | 0.4536 | 0.0477 | 0.0093 | 0.9997 |
| NM_009260.2 | *Spnb2* | Mus musculus spectrin beta 2 (Spnb2), transcript variant 2, mRNA. | 0.9922 | 0.0415 | 0.0109 | 0.9997 |
| XM_142623.3 | *LOC245147* | ILMN_199311 | 0.2211 | 0.0044 | 0.0111 | 0.9997 |
| NM_175490.3 | *Gpr75* | Mus musculus G protein-coupled receptor 75 (Gpr75), mRNA. | 0.8552 | 0.0342 | 0.0116 | 0.9997 |
| NM_025723.2 | *4921515J06Rik* | Mus musculus RIKEN cDNA 4921515J06 gene (4921515J06Rik), transcript variant 1, mRNA. | 0.5206 | 0.0277 | 0.0123 | 0.9997 |
| NM_145391.1 | *Tapbpl* | Mus musculus TAP binding protein-like (Tapbpl), mRNA. | 0.7822 | 0.0244 | 0.0125 | 0.9997 |
| NM_022326.2 | *Ctsm* | Mus musculus cathepsin M (Ctsm), mRNA. | 0.4311 | 0.0056 | 0.0125 | 0.9997 |
| NM_016919.1 | *Col5a3* |  | 0.1794 | 0.0112 | 0.0129 | 0.9997 |
| XM_207109.3 | *LOC280096* | ILMN_198060 | 0.6868 | 0.0283 | 0.0146 | 0.9997 |
| XM_355613.1 | *LOC381650* | ILMN_200090 | 0.7647 | 0.0343 | 0.0152 | 0.9997 |
| XM_136701.2 | *LOC226955* | ILMN_197128 | 0.8249 | 0.0215 | 0.0154 | 0.9997 |
| NM_020000.2 | *Med8* | Mus musculus mediator of RNA polymerase II transcription, subunit 8 homolog (yeast) (Med8), transcript variant 1, mRNA. | 0.8031 | 0.0384 | 0.0163 | 0.9997 |
| NM_001001983.1 | *Pi4ka* | Mus musculus phosphatidylinositol 4-kinase, catalytic, alpha polypeptide (Pi4ka), mRNA. | 0.8574 | 0.0264 | 0.0164 | 0.9997 |
| XM_130322 | *Ttn* |  | 0.5446 | 0.0165 | 0.0166 | 0.9997 |
| NM_053176.1 | *Hrg* | Mus musculus histidine-rich glycoprotein (Hrg), mRNA. | 0.9954 | 0.0076 | 0.0169 | 0.9997 |
|  | *MJ-1000-56_266* | ILMN_194618 | 0.6639 | 0.0221 | 0.0178 | 0.9997 |
| AK014396 | *3632411M23Rik* | ILMN_202249 | 0.1251 | 0.0171 | 0.0181 | 0.9997 |
| NM_146151.3 | *Tesk2* | Mus musculus testis-specific kinase 2 (Tesk2), mRNA. | 0.3443 | 0.0493 | 0.0220 | 0.9997 |
| NM_023750.2 | *Zfp84* | Mus musculus zinc finger protein 84 (Zfp84), mRNA. | 0.4237 | 0.0079 | 0.0234 | 0.9997 |
| XM_132218.3 | *2310002F18Rik* | ILMN_219267 | 0.2586 | 0.0395 | 0.0264 | 0.9997 |
| NM_001001295.1 | *Dis3l* | Mus musculus DIS3 mitotic control homolog (S. cerevisiae)-like (Dis3l), transcript variant 1, mRNA. | 0.4166 | 0.0234 | 0.0272 | 0.9997 |
| AK079826 | *A430077H15Rik* | ILMN_206138 | 0.5091 | 0.0358 | 0.0274 | 0.9997 |
| NM_001033540.2 | *EG330503* | Mus musculus predicted gene, EG330503 (EG330503), mRNA. | 0.7685 | 0.0115 | 0.0276 | 0.9997 |
| NM_010763.1 | *Man1a2* | Mus musculus mannosidase, alpha, class 1A, member 2 (Man1a2), mRNA. | 0.2493 | 0.0274 | 0.0279 | 0.9997 |
| NM_198031.1 | *Tubgcp3* | Mus musculus tubulin, gamma complex associated protein 3 (Tubgcp3), mRNA. | 0.1358 | 0.0028 | 0.0288 | 0.9997 |
| AK006776 | *1700052N19Rik* | ILMN_202189 | 0.4641 | 0.0100 | 0.0295 | 0.9997 |
| XM_914689.2 | *Slc6a16* | PREDICTED: Mus musculus solute carrier family 6, member 16 (Slc6a16), mRNA. | 0.5782 | 0.0202 | 0.0302 | 0.9997 |
| NM_011841.1 | *Mapk7* | Mus musculus mitogen-activated protein kinase 7 (Mapk7), mRNA. | 0.6779 | 0.0486 | 0.0304 | 0.9997 |
| NM_011776.1 | *Zp3* | Mus musculus zona pellucida glycoprotein 3 (Zp3), mRNA. | 0.5951 | 0.0492 | 0.0307 | 0.9997 |
| NM_023773.1 | *Mphosph8* | Mus musculus M-phase phosphoprotein 8 (Mphosph8), mRNA. | 0.3360 | 0.0005 | 0.0314 | 0.9997 |
| AK051805 | *D130099D04Rik* | ILMN_204932 | 0.7036 | 0.0488 | 0.0319 | 0.9997 |
| NM_028965.3 | *Snx11* | Mus musculus sorting nexin 11 (Snx11), mRNA. | 0.9572 | 0.0483 | 0.0325 | 0.9997 |
| NM_134000.3 | *Traf3ip2* | Mus musculus Traf3 interacting protein 2 (Traf3ip2), mRNA. | 0.4632 | 0.0323 | 0.0329 | 0.9997 |
| AK086629 | *D930042N17Rik* | ILMN_206534 | 0.9531 | 0.0003 | 0.0334 | 0.9997 |
| BC004695 | *Zfp64* |  | 0.6911 | 0.0009 | 0.0336 | 0.9997 |
| NM_001001182.3 | *Baz2b* | Mus musculus bromodomain adjacent to zinc finger domain, 2B (Baz2b), mRNA. | 0.3754 | 0.0222 | 0.0344 | 0.9997 |
| NM_028990.2 | *Tmem168* | Mus musculus transmembrane protein 168 (Tmem168), mRNA. | 0.3043 | 0.0419 | 0.0346 | 0.9997 |
| NM_133194.3 | *Scml2* | Mus musculus sex comb on midleg-like 2 (Drosophila) (Scml2), mRNA. | 0.4546 | 0.0446 | 0.0355 | 0.9997 |
|  | *IGKV2-112_J00562_Ig_kappa_variable_2-112_55* | ILMN_185204 | 0.6577 | 0.0028 | 0.0358 | 0.9997 |
| NM_146795.1 | *Olfr812* | Mus musculus olfactory receptor 812 (Olfr812), mRNA. | 0.1444 | 0.0450 | 0.0370 | 0.9997 |
| NM_173769.3 | *Zfp641* | Mus musculus zinc finger protein 641 (Zfp641), mRNA. | 0.8946 | 0.0257 | 0.0372 | 0.9997 |
| NM_001048057.1 | *Rpl38* | Mus musculus ribosomal protein L38 (Rpl38), transcript variant 1, mRNA. | 0.4074 | 0.0465 | 0.0373 | 0.9997 |
| NM_207528 | *5330416C01Rik* | ILMN_219092 | 0.9076 | 0.0493 | 0.0376 | 0.9997 |
| NM_010267.2 | *Gdap1* | Mus musculus ganglioside-induced differentiation-associated-protein 1 (Gdap1), mRNA. | 0.2808 | 0.0385 | 0.0376 | 0.9997 |
| NM_172124.2 | *B3gat2* | Mus musculus beta-1,3-glucuronyltransferase 2 (glucuronosyltransferase S) (B3gat2), mRNA. | 0.9568 | 0.0320 | 0.0377 | 0.9997 |
| NM_172841 | *Slco5a1* |  | 0.3819 | 0.0352 | 0.0393 | 0.9997 |
| NM_030262.3 | *Pofut2* | Mus musculus protein O-fucosyltransferase 2 (Pofut2), mRNA. | 0.5354 | 0.0092 | 0.0409 | 0.9997 |
| NM_001033263.1 | *Centg1* | Mus musculus centaurin, gamma 1 (Centg1), mRNA. | 0.6844 | 0.0021 | 0.0421 | 0.9997 |
| XM_001473773.1 | *LOC631071* | PREDICTED: Mus musculus similar to RIKEN cDNA 2610208M17 gene (LOC631071), mRNA. | 0.5776 | 0.0498 | 0.0423 | 0.9997 |
| XM_621774.1 | *EG546954* | PREDICTED: Mus musculus predicted gene, EG546954 (EG546954), mRNA. | 0.3100 | 0.0245 | 0.0439 | 0.9997 |
| NM_175229.3 | *Srrm2* | Mus musculus serine/arginine repetitive matrix 2 (Srrm2), mRNA. | 0.3153 | 0.0209 | 0.0442 | 0.9997 |
| AK054364 | *E330019I03Rik* | ILMN_205263 | 0.2546 | 0.0402 | 0.0444 | 0.9997 |
| NM_027373.2 | *Afap1* | Mus musculus actin filament associated protein 1 (Afap1), mRNA. | 0.6358 | 0.0024 | 0.0448 | 0.9997 |
| XM_359179 | *1700008H02Rik* | ILMN_188746 | 0.8776 | 0.0090 | 0.0450 | 0.9997 |
| NM_175497.3 | *Actbl2* | Mus musculus actin, beta-like 2 (Actbl2), mRNA. | 0.9448 | 0.0474 | 0.0476 | 0.9997 |
| NM_080728.2 | *Myh7* | Mus musculus myosin, heavy polypeptide 7, cardiac muscle, beta (Myh7), mRNA. | 0.2051 | 0.0431 | 0.0478 | 0.9997 |
| NM_001033434.1 | *Gm884* | Mus musculus gene model 884, (NCBI) (Gm884), mRNA. | 0.6174 | 0.0229 | 0.0493 | 0.9997 |
| NM_008812.1 | *Padi2* | Mus musculus peptidyl arginine deiminase, type II (Padi2), mRNA. | 0.6709 | 0.0147 | 0.0493 | 0.9997 |

FDR, false discovery rate using a Benjamini and Hochberg multiple testing correction.
