# Supplementary material for: Genome-wide analysis identifies colonic genes differentially associated with serum leptin and insulin concentrations in C57BL/6J mice fed a high-fat diet
Source: PLoS One. 2017 Feb 7;12(2):e0171664. doi: 10.1371/journal.pone.0171664 (PMC5295695; doi:10.1371/journal.pone.0171664)
Supplement: S5 Table — (DOCX) [file pone.0171664.s005.docx]

**S5 Table. List of differentially expressed and serum adiponectin-associated genes in the colon tissue of high-fat diet fed C57BL/J mice.**

1. Inverse relationships between two dietary groups

| Accession | Symbol | Definition | P-value (interaction; M_PAG1_) | FDR  (interaction) | P-value (t-test; M_DEG_) | FDR  (t-test) |
| --- | --- | --- | --- | --- | --- | --- |
| NM_027922.1 | *Ankle2* | Mus musculus ankyrin repeat and LEM domain containing 2 (Ankle2), mRNA. | 0.0158 | 0.2567 | 0.0001 | 0.9321 |
| NM_001033394.1 | *A230069A22Rik* | Mus musculus RIKEN cDNA A230069A22 gene (A230069A22Rik), mRNA. | 0.0207 | 0.2567 | 0.0002 | 0.9321 |
| XM_001472820.1 | *LOC100039516* | PREDICTED: Mus musculus similar to putative, transcript variant 1 (LOC100039516), mRNA. | 0.0201 | 0.2567 | 0.0004 | 0.9321 |
| NM_001007579.1 | *BC080695* | Mus musculus cDNA sequence BC080695 (BC080695), mRNA. | 0.0144 | 0.2567 | 0.0005 | 0.9321 |
| NM_010424.2 | *Hfe* | Mus musculus hemochromatosis (Hfe), mRNA. | 0.0237 | 0.2567 | 0.0007 | 0.9321 |
| NM_027758.3 | *Tbc1d9* | Mus musculus TBC1 domain family, member 9 (Tbc1d9), mRNA. | 0.0107 | 0.2567 | 0.0007 | 0.9321 |
| NM_146967.1 | *Olfr1226* | Mus musculus olfactory receptor 1226 (Olfr1226), mRNA. | 0.0171 | 0.2567 | 0.0007 | 0.9321 |
| NM_029588.1 | *1700012L04Rik* | Mus musculus RIKEN cDNA 1700012L04 gene (1700012L04Rik), mRNA. | 0.0269 | 0.2567 | 0.0008 | 0.9321 |
| NM_133889.2 | *Bsdc1* | Mus musculus BSD domain containing 1 (Bsdc1), mRNA. | 0.0250 | 0.2567 | 0.0008 | 0.9321 |
| NM_028848.2 | *Spata17* | Mus musculus spermatogenesis associated 17 (Spata17), mRNA. | 0.0320 | 0.2567 | 0.0009 | 0.9321 |
| NM_138673.2 | *Stab2* | Mus musculus stabilin 2 (Stab2), mRNA. | 0.0492 | 0.2589 | 0.0010 | 0.9321 |
| AK013777 | *Ptpn21* |  | 0.0429 | 0.2569 | 0.0019 | 0.9321 |
| NM_175086.2 | *Agtr1b* | Mus musculus angiotensin II receptor, type 1b (Agtr1b), mRNA. | 0.0256 | 0.2567 | 0.0021 | 0.9321 |
| NM_146855 | *Olfr985* |  | 0.0012 | 0.2567 | 0.0023 | 0.9321 |
| NM_023456.2 | *Npy* | Mus musculus neuropeptide Y (Npy), mRNA. | 0.0232 | 0.2567 | 0.0026 | 0.9321 |
| NM_019823.3 | *Cyp2d22* | Mus musculus cytochrome P450, family 2, subfamily d, polypeptide 22 (Cyp2d22), mRNA. | 0.0198 | 0.2567 | 0.0027 | 0.9321 |
| NM_007706.3 | *Socs2* | Mus musculus suppressor of cytokine signaling 2 (Socs2), mRNA. | 0.0188 | 0.2567 | 0.0028 | 0.9321 |
| NM_177806.2 | *Prpf39* | Mus musculus PRP39 pre-mRNA processing factor 39 homolog (yeast) (Prpf39), mRNA. | 0.0173 | 0.2567 | 0.0028 | 0.9321 |
| XR_035305.1 | *1700009J07Rik* | PREDICTED: Mus musculus RIKEN cDNA 1700009J07 gene (1700009J07Rik), misc RNA. | 0.0205 | 0.2567 | 0.0030 | 0.9372 |
| NM_027151.1 | *Dctn2* | Mus musculus dynactin 2 (Dctn2), mRNA. | 0.0264 | 0.2567 | 0.0030 | 0.9383 |
| XM_357535.1 | *LOC384276* | ILMN_200423 | 0.0352 | 0.2567 | 0.0030 | 0.9383 |
| NM_134179.1 | *V1rc24* | Mus musculus vomeronasal 1 receptor, C24 (V1rc24), mRNA. | 0.0139 | 0.2567 | 0.0033 | 0.9659 |
| AK081222 | *C030011I11Rik* | ILMN_207205 | 0.0158 | 0.2567 | 0.0034 | 0.9659 |
| NM_026455.2 | *2510049I19Rik* | Mus musculus RIKEN cDNA 2510049I19 gene (2510049I19Rik), mRNA. | 0.0217 | 0.2567 | 0.0039 | 0.9659 |
| NM_053207.1 | *Egln1* | Mus musculus EGL nine homolog 1 (C. elegans) (Egln1), mRNA. | 0.0277 | 0.2567 | 0.0039 | 0.9659 |
| NM_031379.2 | *Tktl1* | Mus musculus transketolase-like 1 (Tktl1), mRNA. | 0.0458 | 0.2571 | 0.0040 | 0.9659 |
| NM_010431.1 | *Hif1a* | Mus musculus hypoxia inducible factor 1, alpha subunit (Hif1a), mRNA. | 0.0082 | 0.2567 | 0.0041 | 0.9659 |
| NM_001034878.2 | *Dnaic2* | Mus musculus dynein, axonemal, intermediate chain 2 (Dnaic2), mRNA. | 0.0014 | 0.2567 | 0.0041 | 0.9659 |
| NM_027196 | *Pold4* |  | 0.0110 | 0.2567 | 0.0042 | 0.9681 |
| XR_032231.1 | *LOC100040863* | PREDICTED: Mus musculus hypothetical protein LOC100040863 (LOC100040863), misc RNA. | 0.0080 | 0.2567 | 0.0046 | 0.9917 |
| NM_001002896.2 | *Bfsp2* | Mus musculus beaded filament structural protein 2, phakinin (Bfsp2), mRNA. | 0.0161 | 0.2567 | 0.0048 | 0.9978 |
| NM_008539.3 | *Smad1* | Mus musculus MAD homolog 1 (Drosophila) (Smad1), mRNA. | 0.0297 | 0.2567 | 0.0052 | 0.9998 |
| NM_176842.2 | *Tprkb* | Mus musculus Tp53rk binding protein (Tprkb), mRNA. | 0.0343 | 0.2567 | 0.0055 | 0.9998 |
| AK083226 | *4921507O14Rik* | ILMN_206123 | 0.0397 | 0.2567 | 0.0055 | 0.9998 |
| AK089490 | *F730039D13Rik* | ILMN_207163 | 0.0158 | 0.2567 | 0.0058 | 0.9998 |
| XR_033756.1 | *LOC100047935* | PREDICTED: Mus musculus similar to ribosomal protein L5 (LOC100047935), misc RNA. | 0.0445 | 0.2569 | 0.0059 | 0.9998 |
| NM_031194.3 | *Slc22a8* | Mus musculus solute carrier family 22 (organic anion transporter), member 8 (Slc22a8), mRNA. | 0.0306 | 0.2567 | 0.0061 | 0.9998 |
| NM_145425.3 | *AV249152* | Mus musculus expressed sequence AV249152 (AV249152), mRNA. | 0.0283 | 0.2567 | 0.0061 | 0.9998 |
| NM_001013380.1 | *Dync1li2* | Mus musculus dynein, cytoplasmic 1 light intermediate chain 2 (Dync1li2), mRNA. | 0.0194 | 0.2567 | 0.0063 | 0.9998 |
| NM_001081383.1 | *Mll3* | Mus musculus myeloid/lymphoid or mixed-lineage leukemia 3 (Mll3), mRNA. | 0.0052 | 0.2567 | 0.0067 | 0.9998 |
| AK050316 | *C730036N12Rik* | ILMN_204165 | 0.0343 | 0.2567 | 0.0067 | 0.9998 |
| AK051959 | *Fap* |  | 0.0162 | 0.2567 | 0.0068 | 0.9998 |
| AK041965 | *A630049E09Rik* | ILMN_203995 | 0.0487 | 0.2589 | 0.0068 | 0.9998 |
| NM_199068.2 | *Foxk1* | Mus musculus forkhead box K1 (Foxk1), mRNA. | 0.0166 | 0.2567 | 0.0069 | 0.9998 |
| NM_001081292.1 | *Map3k10* | Mus musculus mitogen-activated protein kinase kinase kinase 10 (Map3k10), mRNA. | 0.0358 | 0.2567 | 0.0070 | 0.9998 |
| NM_153510.1 | *Pilra* | Mus musculus paired immunoglobin-like type 2 receptor alpha (Pilra), mRNA. | 0.0491 | 0.2589 | 0.0070 | 0.9998 |
| NM_031168.1 | *Il6* | Mus musculus interleukin 6 (Il6), mRNA. | 0.0208 | 0.2567 | 0.0071 | 0.9998 |
| NM_147060 | *Olfr667* |  | 0.0117 | 0.2567 | 0.0071 | 0.9998 |
| NM_001033820.2 | *Zfp551* | Mus musculus zinc fingr protein 551 (Zfp551), mRNA. XM_921380 XM_921386 XM_921391 XM_921394 | 0.0137 | 0.2567 | 0.0073 | 0.9998 |
| XR_033273.1 | *LOC668993* | PREDICTED: Mus musculus similar to casein kinase II alpha subunit (LOC668993), misc RNA. | 0.0168 | 0.2567 | 0.0074 | 0.9998 |
| NM_013541.1 | *Gstp1* | Mus musculus glutathione S-transferase, pi 1 (Gstp1), mRNA. | 0.0150 | 0.2567 | 0.0075 | 0.9998 |
| NM_146477.1 | *Olfr90* | Mus musculus olfactory receptor 90 (Olfr90), mRNA. | 0.0463 | 0.2574 | 0.0079 | 0.9998 |
| NM_145431.1 | *Nle1* | Mus musculus notchless homolog 1 (Drosophila) (Nle1), mRNA. | 0.0057 | 0.2567 | 0.0080 | 0.9998 |
| XR_003429.1 | *LOC671453* | PREDICTED: Mus musculus hypothetical protein LOC671453 (LOC671453), mRNA. | 0.0283 | 0.2567 | 0.0084 | 0.9998 |
| NM_010749.6 | *M6pr* | Mus musculus mannose-6-phosphate receptor, cation dependent (M6pr), mRNA. | 0.0427 | 0.2569 | 0.0085 | 0.9998 |
| NM_027930.1 | *2610016C23Rik* | Mus musculus RIKEN cDNA 2610016C23 gene (2610016C23Rik), mRNA. | 0.0058 | 0.2567 | 0.0088 | 0.9998 |
| NM_001005485.1 | *Olfr111* | Mus musculus olfactory receptor 111 (Olfr111), mRNA. | 0.0468 | 0.2577 | 0.0092 | 0.9998 |
| XR_004802.1 | *LOC675565* | PREDICTED: Mus musculus hypothetical protein LOC675565 (LOC675565), mRNA. | 0.0179 | 0.2567 | 0.0092 | 0.9998 |
| NM_021498.2 | *Pole3* | Mus musculus polymerase (DNA directed), epsilon 3 (p17 subunit) (Pole3), mRNA. | 0.0254 | 0.2567 | 0.0092 | 0.9998 |
| NM_145993.2 | *L3mbtl2* | Mus musculus l(3)mbt-like 2 (Drosophila) (L3mbtl2), mRNA. | 0.0176 | 0.2567 | 0.0093 | 0.9998 |
| XM_001478921.1 | *LOC100047816* | PREDICTED: Mus musculus similar to Dnase1l2 protein (LOC100047816), mRNA. | 0.0219 | 0.2567 | 0.0094 | 0.9998 |
| NM_146990.1 | *Olfr1494* | Mus musculus olfactory receptor 1494 (Olfr1494), mRNA. | 0.0213 | 0.2567 | 0.0097 | 0.9998 |
| NM_001033211.1 | *AU022751* | Mus musculus expressed sequence AU022751 (AU022751), mRNA. | 0.0168 | 0.2567 | 0.0099 | 0.9998 |
| NM_033078.3 | *Klrk1* | Mus musculus killer cell lectin-like receptor subfamily K, member 1 (Klrk1), transcript variant 1, mRNA. | 0.0237 | 0.2567 | 0.0103 | 0.9998 |
| XR_004731.1 | *LOC675098* | PREDICTED: Mus musculus similar to jumonji domain containing 2D (LOC675098), mRNA. | 0.0196 | 0.2567 | 0.0104 | 0.9998 |
| XM_001475886.1 | *LOC100046255* | PREDICTED: Mus musculus similar to homeobox protein (LOC100046255), mRNA. | 0.0340 | 0.2567 | 0.0104 | 0.9998 |
| NM_012027 | *AA536749* | ILMN_186130 | 0.0205 | 0.2567 | 0.0106 | 0.9998 |
| NM_023058.2 | *Pkmyt1* | Mus musculus protein kinase, membrane associated tyrosine/threonine 1 (Pkmyt1), mRNA. | 0.0243 | 0.2567 | 0.0109 | 0.9998 |
| NM_027978.1 | *Coq2* | Mus musculus coenzyme Q2 homolog, prenyltransferase (yeast) (Coq2), mRNA. | 0.0387 | 0.2567 | 0.0109 | 0.9998 |
| NM_013836.2 | *Tcf20* | Mus musculus transcription factor 20 (Tcf20), mRNA. | 0.0179 | 0.2567 | 0.0110 | 0.9998 |
| AK043258 | *Zfp276* |  | 0.0061 | 0.2567 | 0.0111 | 0.9998 |
| NM_175091.3 | *Tnks* | Mus musculus tankyrase, TRF1-interacting ankyrin-related ADP-ribose polymerase (Tnks), mRNA. | 0.0368 | 0.2567 | 0.0112 | 0.9998 |
| NM_001025156.1 | *Ccdc93* | Mus musculus coiled-coil domain containing 93 (Ccdc93), transcript variant 1, mRNA. | 0.0294 | 0.2567 | 0.0113 | 0.9998 |
| NM_177727.2 | *Lsm14b* | Mus musculus LSM14 homolog B (SCD6, S. cerevisiae) (Lsm14b), mRNA. | 0.0496 | 0.2593 | 0.0113 | 0.9998 |
| NM_030706.1 | *Trim2* | Mus musculus tripartite motif protein 2 (Trim2), mRNA. XM_984114 XM_984144 XM_984172 XM_984200 XM_984238 XM_984275 XM_984313 | 0.0202 | 0.2567 | 0.0115 | 0.9998 |
| XM_137322.8 | *4930505D03Rik* | PREDICTED: Mus musculus RIKEN cDNA 4930505D03 gene (4930505D03Rik), mRNA. | 0.0477 | 0.2584 | 0.0117 | 0.9998 |
| NM_028647.1 | *Spata3* | Mus musculus spermatogenesis associated 3 (Spata3), transcript variant 3, mRNA. | 0.0188 | 0.2567 | 0.0118 | 0.9998 |
| NM_026799.2 | *Rnasen* | Mus musculus ribonuclease III, nuclear (Rnasen), mRNA. | 0.0076 | 0.2567 | 0.0118 | 0.9998 |
| NM_007500.3 | *Atoh1* | Mus musculus atonal homolog 1 (Drosophila) (Atoh1), mRNA. | 0.0155 | 0.2567 | 0.0120 | 0.9998 |
| NM_029440.3 | *4930434E21Rik* | Mus musculus RIKEN cDNA 4930434E21 gene (4930434E21Rik), mRNA. | 0.0297 | 0.2567 | 0.0121 | 0.9998 |
| NM_183204.1 | *Rnf182* | Mus musculus ring finger protein 182 (Rnf182), mRNA. | 0.0120 | 0.2567 | 0.0124 | 0.9998 |
| NM_026069.2 | *Rpl37* | Mus musculus ribosomal protein L37 (Rpl37), mRNA. | 0.0417 | 0.2569 | 0.0126 | 0.9998 |
| NM_011438 | *Sox12* |  | 0.0206 | 0.2567 | 0.0128 | 0.9998 |
| NM_001024706.1 | *EG432825* | Mus musculus predicted gene, EG432825 (EG432825), mRNA. | 0.0256 | 0.2567 | 0.0128 | 0.9998 |
| NM_146351.1 | *Olfr1133* | Mus musculus olfactory receptor 1133 (Olfr1133), mRNA. | 0.0434 | 0.2569 | 0.0129 | 0.9998 |
| NM_008539.3 | *Smad1* | Mus musculus MAD homolog 1 (Drosophila) (Smad1), mRNA. | 0.0026 | 0.2567 | 0.0133 | 0.9998 |
| AK046579 | *Cmkbr2* | ILMN_203489 | 0.0256 | 0.2567 | 0.0133 | 0.9998 |
| AK029049 | *4732485D01Rik* | ILMN_202924 | 0.0095 | 0.2567 | 0.0133 | 0.9998 |
| NM_025978.3 | *Ttc14* | Mus musculus tetratricopeptide repeat domain 14 (Ttc14), transcript variant 2, mRNA. | 0.0026 | 0.2567 | 0.0135 | 0.9998 |
| AK043011 | *A730047M10Rik* | ILMN_204294 | 0.0204 | 0.2567 | 0.0137 | 0.9998 |
| NM_027186.1 | *Rpain* | Mus musculus RPA interacting protein (Rpain), mRNA. | 0.0426 | 0.2569 | 0.0138 | 0.9998 |
| NM_010701.1 | *Lect1* | Mus musculus leukocyte cell derived chemotaxin 1 (Lect1), mRNA. | 0.0041 | 0.2567 | 0.0138 | 0.9998 |
| NM_010949 | *Numb* |  | 0.0276 | 0.2567 | 0.0139 | 0.9998 |
| NM_009060.2 | *Rgn* | Mus musculus regucalcin (Rgn), mRNA. | 0.0167 | 0.2567 | 0.0140 | 0.9998 |
| NM_021279.4 | *Wnt1* | Mus musculus wingless-related MMTV integration site 1 (Wnt1), mRNA. | 0.0490 | 0.2589 | 0.0140 | 0.9998 |
|  | *1110021P09Rik* | ILMN_187053 | 0.0225 | 0.2567 | 0.0142 | 0.9998 |
| NM_011449.1 | *Spa17* | Mus musculus sperm autoantigenic protein 17 (Spa17), mRNA. | 0.0113 | 0.2567 | 0.0143 | 0.9998 |
| NM_010212.3 | *Fhl2* | Mus musculus four and a half LIM domains 2 (Fhl2), mRNA. | 0.0252 | 0.2567 | 0.0143 | 0.9998 |
| XM_001480668.1 | *ENSMUSG00000044227* | PREDICTED: Mus musculus predicted gene, ENSMUSG00000044227 (ENSMUSG00000044227), mRNA. | 0.0474 | 0.2581 | 0.0143 | 0.9998 |
| XM_989242.1 | *Zfp508* | PREDICTED: Mus musculus zinc finger protein 508 (Zfp508), mRNA. | 0.0120 | 0.2567 | 0.0144 | 0.9998 |
| XM_123302.2 | *LOC225763* | ILMN_198268 | 0.0126 | 0.2567 | 0.0144 | 0.9998 |
| NM_010325 | *Got2* |  | 0.0313 | 0.2567 | 0.0144 | 0.9998 |
| NM_010137.2 | *Epas1* | Mus musculus endothelial PAS domain protein 1 (Epas1), mRNA. | 0.0351 | 0.2567 | 0.0149 | 0.9998 |
| NM_172835.2 | *Peli3* | Mus musculus pellino 3 (Peli3), mRNA. | 0.0102 | 0.2567 | 0.0153 | 0.9998 |
| NM_145940.2 | *Wipi1* | Mus musculus WD repeat domain, phosphoinositide interacting 1 (Wipi1), mRNA. | 0.0181 | 0.2567 | 0.0155 | 0.9998 |
| XM_357434.1 | *LOC384112* | ILMN_199962 | 0.0152 | 0.2567 | 0.0157 | 0.9998 |
| NM_144946.3 | *Neto1* | Mus musculus neuropilin (NRP) and tolloid (TLL)-like 1 (Neto1), mRNA. | 0.0213 | 0.2567 | 0.0158 | 0.9998 |
| XM_485125 | *Etohi1* |  | 0.0126 | 0.2567 | 0.0159 | 0.9998 |
| NM_001011769.1 | *Olfr317* | Mus musculus olfactory receptor 317 (Olfr317), mRNA. | 0.0399 | 0.2567 | 0.0159 | 0.9998 |
| XM_358413.1 | *LOC380731* | ILMN_199219 | 0.0181 | 0.2567 | 0.0160 | 0.9998 |
| XM_144896.1 | *LOC243537* | ILMN_196793 | 0.0262 | 0.2567 | 0.0161 | 0.9998 |
| NM_027196.1 | *Pold4* | Mus musculus polymerase (DNA-directed), delta 4 (Pold4), mRNA. | 0.0141 | 0.2567 | 0.0162 | 0.9998 |
| AK076359 | *4732465J04Rik* | ILMN_207047 | 0.0196 | 0.2567 | 0.0164 | 0.9998 |
| XM_359238.1 | *LOC385642* | ILMN_200149 | 0.0391 | 0.2567 | 0.0164 | 0.9998 |
| AK035512 | *9530058K19Rik* | ILMN_203680 | 0.0138 | 0.2567 | 0.0166 | 0.9998 |
| NM_008048.2 | *Igfbp7* | Mus musculus insulin-like growth factor binding protein 7 (Igfbp7), mRNA. | 0.0082 | 0.2567 | 0.0167 | 0.9998 |
| AK032746 | *6720426O10Rik* | ILMN_203149 | 0.0223 | 0.2567 | 0.0168 | 0.9998 |
| NM_009157.4 | *Map2k4* | Mus musculus mitogen-activated protein kinase kinase 4 (Map2k4), mRNA. | 0.0270 | 0.2567 | 0.0168 | 0.9998 |
| NM_001011828.1 | *Olfr304* | Mus musculus olfactory receptor 304 (Olfr304), mRNA. | 0.0150 | 0.2567 | 0.0170 | 0.9998 |
| NM_013875.2 | *Pde7b* | Mus musculus phosphodiesterase 7B (Pde7b), mRNA. | 0.0092 | 0.2567 | 0.0173 | 0.9998 |
| AK044324 | *A930007K04Rik* | ILMN_203050 | 0.0020 | 0.2567 | 0.0174 | 0.9998 |
| NM_198664.3 | *Tbc1d2* | Mus musculus TBC1 domain family, member 2 (Tbc1d2), mRNA. | 0.0343 | 0.2567 | 0.0174 | 0.9998 |
| NM_146853.2 | *Olfr1341* | Mus musculus olfactory receptor 1341 (Olfr1341), mRNA. | 0.0488 | 0.2589 | 0.0174 | 0.9998 |
| NM_001081012.1 | *4930473A06Rik* | Mus musculus RIKEN cDNA 4930473A06 gene (4930473A06Rik), mRNA. | 0.0263 | 0.2567 | 0.0175 | 0.9998 |
| NM_133852.2 | *Golga2* | Mus musculus golgi autoantigen, golgin subfamily a, 2 (Golga2), transcript variant 1, mRNA. XM_917195 XM_924742 XM_924744 XM_924745 XM_924752 XM_924758 XM_990733 XM_990778 XM_990810 XM_990841 XM_990863 | 0.0032 | 0.2567 | 0.0177 | 0.9998 |
| NM_011563.2 | *Prdx2* | Mus musculus peroxiredoxin 2 (Prdx2), mRNA. | 0.0242 | 0.2567 | 0.0177 | 0.9998 |
| AK029211 | *4831422N07Rik* | ILMN_203457 | 0.0213 | 0.2567 | 0.0180 | 0.9998 |
|  | *A630036G19Rik* | ILMN_190850 | 0.0481 | 0.2587 | 0.0181 | 0.9998 |
| NM_174865.1 | *Klk15* | Mus musculus kallikrein related-peptidase 15 (Klk15), mRNA. | 0.0289 | 0.2567 | 0.0186 | 0.9998 |
| NM_009021.2 | *Rai1* | Mus musculus retinoic acid induced 1 (Rai1), transcript variant 1, mRNA. | 0.0403 | 0.2567 | 0.0187 | 0.9998 |
| NM_028037.3 | *Acad10* | Mus musculus acyl-Coenzyme A dehydrogenase family, member 10 (Acad10), mRNA. | 0.0310 | 0.2567 | 0.0188 | 0.9998 |
| AK045099 | *B130032G09Rik* | ILMN_205347 | 0.0452 | 0.2569 | 0.0190 | 0.9998 |
| NM_144910 | *Cnot6l* |  | 0.0149 | 0.2567 | 0.0191 | 0.9998 |
| NM_146490.1 | *Olfr1411* | Mus musculus olfactory receptor 1411 (Olfr1411), mRNA. | 0.0157 | 0.2567 | 0.0193 | 0.9998 |
| XM_001476947.1 | *LOC100041754* | PREDICTED: Mus musculus hypothetical protein LOC100041754 (LOC100041754), mRNA. | 0.0383 | 0.2567 | 0.0194 | 0.9998 |
| NM_178139.1 | *Otop1* | Mus musculus otopetrin 1 (Otop1), transcript variant b, mRNA. | 0.0487 | 0.2589 | 0.0194 | 0.9998 |
| XR_030810.1 | *LOC666036* | PREDICTED: Mus musculus similar to 3-phosphoglycerate dehydrogenase (LOC666036), misc RNA. | 0.0084 | 0.2567 | 0.0195 | 0.9998 |
| AK035619 | *Mapk8ip3* |  | 0.0351 | 0.2567 | 0.0197 | 0.9998 |
| XM_358066.1 | *LOC385117* | ILMN_201411 | 0.0365 | 0.2567 | 0.0198 | 0.9998 |
| NM_145353.1 | *Yipf3* | Mus musculus Yip1 domain family, member 3 (Yipf3), mRNA. | 0.0181 | 0.2567 | 0.0199 | 0.9998 |
| NM_028533.2 | *1700065D16Rik* | Mus musculus RIKEN cDNA 1700065D16 gene (1700065D16Rik), mRNA. | 0.0339 | 0.2567 | 0.0201 | 0.9998 |
| XM_354553.1 | *LOC380644* | ILMN_199047 | 0.0285 | 0.2567 | 0.0201 | 0.9998 |
| NM_001011832.1 | *Olfr1490* | Mus musculus olfactory receptor 1490 (Olfr1490), mRNA. | 0.0232 | 0.2567 | 0.0203 | 0.9998 |
| NM_001033157.3 | *5730507C01Rik* | Mus musculus RIKEN cDNA 5730507C01 gene (5730507C01Rik), mRNA. | 0.0187 | 0.2567 | 0.0204 | 0.9998 |
| XM_356873.1 | *LOC383099* | ILMN_200013 | 0.0265 | 0.2567 | 0.0204 | 0.9998 |
| NM_178668 | *E430028B21Rik* | ILMN_186023 | 0.0088 | 0.2567 | 0.0208 | 0.9998 |
| NM_175229.3 | *Srrm2* | Mus musculus serine/arginine repetitive matrix 2 (Srrm2), mRNA. | 0.0264 | 0.2567 | 0.0209 | 0.9998 |
| NM_153802 | *Zfp128* |  | 0.0225 | 0.2567 | 0.0210 | 0.9998 |
| NM_130456.3 | *Nphs2* | Mus musculus nephrosis 2 homolog, podocin (human) (Nphs2), mRNA. | 0.0233 | 0.2567 | 0.0210 | 0.9998 |
| NM_001033529.1 | *2210038L17Rik* | Mus musculus RIKEN cDNA 2210038L17 gene (2210038L17Rik), mRNA. | 0.0105 | 0.2567 | 0.0211 | 0.9998 |
| NM_177713.3 | *Gapt* | Mus musculus Grb2-binding adaptor, transmembrane (Gapt), mRNA. | 0.0398 | 0.2567 | 0.0212 | 0.9998 |
| NM_001025371.1 | *Serinc4* | Mus musculus serine incorporator 4 (Serinc4), mRNA. | 0.0021 | 0.2567 | 0.0213 | 0.9998 |
| AK029350 | *4833401K19Rik* | ILMN_203102 | 0.0011 | 0.2567 | 0.0215 | 0.9998 |
| NM_172303.3 | *Phf17* | Mus musculus PHD finger protein 17 (Phf17), mRNA. | 0.0201 | 0.2567 | 0.0215 | 0.9998 |
| NM_018822.3 | *Sgsh* | Mus musculus N-sulfoglucosamine sulfohydrolase (sulfamidase) (Sgsh), mRNA. | 0.0454 | 0.2569 | 0.0215 | 0.9998 |
| NM_146203.2 | *Zfp764* | Mus musculus zinc finger protein 764 (Zfp764), mRNA. | 0.0211 | 0.2567 | 0.0217 | 0.9998 |
| NM_001008785.3 | *Kbtbd8* | Mus musculus kelch repeat and BTB (POZ) domain containing 8 (Kbtbd8), mRNA. | 0.0319 | 0.2567 | 0.0218 | 0.9998 |
| NM_027897.3 | *Rhpn2* | Mus musculus rhophilin, Rho GTPase binding protein 2 (Rhpn2), mRNA. | 0.0242 | 0.2567 | 0.0222 | 0.9998 |
| NM_144926.2 | *Sez6l2* | Mus musculus seizure related 6 homolog like 2 (Sez6l2), mRNA. | 0.0233 | 0.2567 | 0.0223 | 0.9998 |
| NM_146769 | *Olfr1110* |  | 0.0335 | 0.2567 | 0.0224 | 0.9998 |
| XM_143838.1 | *LOC214444* | ILMN_196795 | 0.0442 | 0.2569 | 0.0226 | 0.9998 |
| NM_011251.2 | *Rbm6* | Mus musculus RNA binding motif protein 6 (Rbm6), transcript variant 1, mRNA. | 0.0165 | 0.2567 | 0.0226 | 0.9998 |
| NM_001039214.3 | *Mex3c* | Mus musculus mex3 homolog C (C. elegans) (Mex3c), mRNA. | 0.0310 | 0.2567 | 0.0228 | 0.9998 |
|  | *1700120C14Rik* | ILMN_186217 | 0.0138 | 0.2567 | 0.0228 | 0.9998 |
| XM_001479450.1 | *Zubr1* | PREDICTED: Mus musculus zinc finger, UBR1 type 1, transcript variant 1 (Zubr1), mRNA. | 0.0452 | 0.2569 | 0.0229 | 0.9998 |
| XR_032154.1 | *LOC100045967* | PREDICTED: Mus musculus hypothetical protein LOC100045967 (LOC100045967), misc RNA. | 0.0221 | 0.2567 | 0.0230 | 0.9998 |
| NM_001038621.2 | *Rabgap1l* | Mus musculus RAB GTPase activating protein 1-like (Rabgap1l), transcript variant 2, mRNA. | 0.0102 | 0.2567 | 0.0230 | 0.9998 |
| XM_354759.1 | *LOC380854* | ILMN_197265 | 0.0215 | 0.2567 | 0.0234 | 0.9998 |
| XM_001471626.1 | *LOC100038857* | PREDICTED: Mus musculus hypothetical protein LOC100038857 (LOC100038857), mRNA. | 0.0116 | 0.2567 | 0.0236 | 0.9998 |
| NM_001039710.1 | *Coq10b* | Mus musculus coenzyme Q10 homolog B (S. cerevisiae) (Coq10b), transcript variant 1, mRNA. | 0.0225 | 0.2567 | 0.0237 | 0.9998 |
| XM_127565 | *Flnb* |  | 0.0013 | 0.2567 | 0.0238 | 0.9998 |
| NM_011498.4 | *Bhlhb2* | Mus musculus basic helix-loop-helix domain containing, class B2 (Bhlhb2), mRNA. | 0.0221 | 0.2567 | 0.0240 | 0.9998 |
| NM_177413 | *B230343H07Rik* | ILMN_185148 | 0.0220 | 0.2567 | 0.0240 | 0.9998 |
| NM_001013412.2 | *Ccl26* | Mus musculus chemokine (C-C motif) ligand 26 (Ccl26), mRNA. | 0.0243 | 0.2567 | 0.0241 | 0.9998 |
| AK012418 | *Rbl1* |  | 0.0342 | 0.2567 | 0.0242 | 0.9998 |
| NM_177084.3 | *Slc9a4* | Mus musculus solute carrier family 9 (sodium/hydrogen exchanger), member 4 (Slc9a4), mRNA. | 0.0054 | 0.2567 | 0.0246 | 0.9998 |
| XM_126005.7 | *Pkd1l1* | PREDICTED: Mus musculus polycystic kidney disease 1 like 1 (Pkd1l1), mRNA. | 0.0054 | 0.2567 | 0.0247 | 0.9998 |
| AK076385 | *4732481D19Rik* | ILMN_207303 | 0.0035 | 0.2567 | 0.0247 | 0.9998 |
| NM_146358.1 | *Olfr677* | Mus musculus olfactory receptor 677 (Olfr677), mRNA. | 0.0137 | 0.2567 | 0.0247 | 0.9998 |
| NM_007631.2 | *Ccnd1* | Mus musculus cyclin D1 (Ccnd1), mRNA. | 0.0269 | 0.2567 | 0.0248 | 0.9998 |
| AK085517.1 | *scl0001416.1_21* | ILMN_188796 | 0.0083 | 0.2567 | 0.0251 | 0.9998 |
| AK006072 | *Skd3* | ILMN_202268 | 0.0379 | 0.2567 | 0.0251 | 0.9998 |
| NM_026703.1 | *Ndufa8* | Mus musculus NADH dehydrogenase (ubiquinone) 1 alpha subcomplex, 8 (Ndufa8), mRNA. | 0.0262 | 0.2567 | 0.0256 | 0.9998 |
| NM_021299.1 | *Ak3* | Mus musculus adenylate kinase 3 (Ak3), nuclear gene encoding mitochondrial protein, mRNA. | 0.0165 | 0.2567 | 0.0256 | 0.9998 |
| NM_008628.2 | *Msh2* | Mus musculus mutS homolog 2 (E. coli) (Msh2), mRNA. | 0.0226 | 0.2567 | 0.0257 | 0.9998 |
| NM_009792.2 | *Camk2a* | Mus musculus calcium/calmodulin-dependent protein kinase II alpha (Camk2a), transcript variant 1, mRNA. | 0.0295 | 0.2567 | 0.0257 | 0.9998 |
| NM_181853.3 | *Trim66* | Mus musculus tripartite motif-containing 66 (Trim66), mRNA. | 0.0454 | 0.2569 | 0.0258 | 0.9998 |
| NM_144889 | *Psmf1* |  | 0.0243 | 0.2567 | 0.0260 | 0.9998 |
| NM_032003.1 | *Enpp5* | Mus musculus ectonucleotide pyrophosphatase/phosphodiesterase 5 (Enpp5), mRNA. | 0.0460 | 0.2571 | 0.0260 | 0.9998 |
| NM_145435.1 | *Pyy* | Mus musculus peptide YY (Pyy), mRNA. | 0.0162 | 0.2567 | 0.0262 | 0.9998 |
| NM_021300.2 | *Rhox4b* | Mus musculus reproductive homeobox 4B (Rhox4b), mRNA. | 0.0228 | 0.2567 | 0.0265 | 0.9998 |
| NM_010574.2 | *Irx2* | Mus musculus Iroquois related homeobox 2 (Drosophila) (Irx2), mRNA. | 0.0158 | 0.2567 | 0.0269 | 0.9998 |
| XM_358997.1 | *LOC385917* | ILMN_200196 | 0.0205 | 0.2567 | 0.0271 | 0.9998 |
| NM_146261.1 | *BC031748* | Mus musculus cDNA sequence BC031748 (BC031748), mRNA. | 0.0298 | 0.2567 | 0.0272 | 0.9998 |
| NM_178732.3 | *Zfp324* | Mus musculus zinc finger protein 324 (Zfp324), mRNA. | 0.0286 | 0.2567 | 0.0273 | 0.9998 |
| XM_359364.1 | *LOC386515* | ILMN_199752 | 0.0392 | 0.2567 | 0.0273 | 0.9998 |
| NM_001081336.1 | *Dgkh* | Mus musculus diacylglycerol kinase, eta (Dgkh), mRNA. | 0.0037 | 0.2567 | 0.0274 | 0.9998 |
| XM_356567.1 | *LOC382556* | ILMN_201368 | 0.0210 | 0.2567 | 0.0278 | 0.9998 |
| AK076679 | *2810021O14Rik* | ILMN_206636 | 0.0166 | 0.2567 | 0.0279 | 0.9998 |
| NM_172450.3 | *4930539E08Rik* | Mus musculus RIKEN cDNA 4930539E08 gene (4930539E08Rik), mRNA. | 0.0278 | 0.2567 | 0.0283 | 0.9998 |
| NM_175347.4 | *Srl* | Mus musculus sarcalumenin (Srl), mRNA. | 0.0346 | 0.2567 | 0.0284 | 0.9998 |
| NM_025735.1 | *Map1lc3a* | Mus musculus microtubule-associated protein 1 light chain 3 alpha (Map1lc3a), mRNA. | 0.0048 | 0.2567 | 0.0287 | 0.9998 |
| XR_034929.1 | *LOC626061* | PREDICTED: Mus musculus similar to E74-like factor 1 (LOC626061), misc RNA. | 0.0475 | 0.2581 | 0.0287 | 0.9998 |
| NM_173047.2 | *Cbr3* | Mus musculus carbonyl reductase 3 (Cbr3), mRNA. | 0.0263 | 0.2567 | 0.0287 | 0.9998 |
| NM_021501.3 | *Pias4* | Mus musculus protein inhibitor of activated STAT 4 (Pias4), mRNA. | 0.0274 | 0.2567 | 0.0287 | 0.9998 |
| AF231926 | *Rabggtb* |  | 0.0259 | 0.2567 | 0.0291 | 0.9998 |
| AK036708 | *9830165F18Rik* | ILMN_203914 | 0.0265 | 0.2567 | 0.0292 | 0.9998 |
| NM_148922.2 | *Mdm1* | Mus musculus transformed mouse 3T3 cell double minute 1 (Mdm1), transcript variant 2, mRNA. | 0.0277 | 0.2567 | 0.0295 | 0.9998 |
| NM_028126.2 | *2610019A05Rik* | Mus musculus RIKEN cDNA 2610019A05 gene (2610019A05Rik), mRNA. | 0.0305 | 0.2567 | 0.0295 | 0.9998 |
| NM_010735.1 | *Lta* | Mus musculus lymphotoxin A (Lta), mRNA. | 0.0126 | 0.2567 | 0.0295 | 0.9998 |
| NM_020016.1 | *Magea2* | Mus musculus melanoma antigen, family A, 2 (Magea2), mRNA. | 0.0151 | 0.2567 | 0.0301 | 0.9998 |
|  | *2810011L19Rik* | ILMN_193236 | 0.0403 | 0.2567 | 0.0302 | 0.9998 |
| NM_026651.1 | *Pomgnt1* | Mus musculus protein O-linked mannose beta1,2-N-acetylglucosaminyltransferase (Pomgnt1), transcript variant 1, mRNA. | 0.0274 | 0.2567 | 0.0302 | 0.9998 |
| XM_906534.2 | *Ccdc121* | PREDICTED: Mus musculus coiled-coil domain containing 121 (Ccdc121), mRNA. | 0.0421 | 0.2569 | 0.0304 | 0.9998 |
| XM_001474081.1 | *LOC383196* | PREDICTED: Mus musculus hypothetical LOC383196 (LOC383196), mRNA. | 0.0131 | 0.2567 | 0.0305 | 0.9998 |
| XM_130929.4 | *LOC229395* | ILMN_197567 | 0.0229 | 0.2567 | 0.0305 | 0.9998 |
| NM_026087.1 | *Ceacam12* | Mus musculus CEA-related cell adhesion molecule 12 (Ceacam12), mRNA. | 0.0310 | 0.2567 | 0.0306 | 0.9998 |
| NM_009477.1 | *Upp1* | Mus musculus uridine phosphorylase 1 (Upp1), mRNA. | 0.0415 | 0.2567 | 0.0306 | 0.9998 |
| NM_177118 | *A830073O21Rik* | ILMN_190551 | 0.0030 | 0.2567 | 0.0307 | 0.9998 |
| NM_025917.3 | *Use1* | Mus musculus unconventional SNARE in the ER 1 homolog (S. cerevisiae) (Use1), transcript variant 1, mRNA. | 0.0191 | 0.2567 | 0.0307 | 0.9998 |
| NM_001035525.1 | *AU040320* | Mus musculus expressed sequence AU040320 (AU040320), transcript variant 2, mRNA. | 0.0482 | 0.2587 | 0.0307 | 0.9998 |
| NM_021451.1 | *Pmaip1* |  | 0.0163 | 0.2567 | 0.0309 | 0.9998 |
| XM_897674.2 | *D14Abb1e* | PREDICTED: Mus musculus DNA segment, Chr 14, Abbott 1 expressed, transcript variant 3 (D14Abb1e), mRNA. | 0.0226 | 0.2567 | 0.0311 | 0.9998 |
| NM_133250.1 | *Mutyh* | Mus musculus mutY homolog (E. coli) (Mutyh), nuclear gene encoding mitochondrial protein, mRNA. | 0.0371 | 0.2567 | 0.0311 | 0.9998 |
| XM_129836 | *Phf3* |  | 0.0343 | 0.2567 | 0.0312 | 0.9998 |
| AK089528 | *F730046H10Rik* | ILMN_207153 | 0.0314 | 0.2567 | 0.0313 | 0.9998 |
| XM_134806.2 | *4931429I11Rik* | ILMN_216350 | 0.0225 | 0.2567 | 0.0313 | 0.9998 |
| NM_029312.1 | *1700010H22Rik* | ILMN_218380 | 0.0247 | 0.2567 | 0.0313 | 0.9998 |
| AK047179 | *Gria4* |  | 0.0235 | 0.2567 | 0.0316 | 0.9998 |
| NM_153053.3 | *Sf3b4* | Mus musculus splicing factor 3b, subunit 4 (Sf3b4), mRNA. | 0.0224 | 0.2567 | 0.0316 | 0.9998 |
| NM_025907.3 | *Mettl6* | Mus musculus methyltransferase like 6 (Mettl6), mRNA. | 0.0434 | 0.2569 | 0.0318 | 0.9998 |
| NM_130458.1 | *Sp7* | Mus musculus trans-acting transcription factor 7 (Sp7), mRNA. | 0.0196 | 0.2567 | 0.0318 | 0.9998 |
| XM_356799.1 | *LOC383012* | ILMN_199786 | 0.0107 | 0.2567 | 0.0324 | 0.9998 |
| XM_287382.1 | *LOC329950* | ILMN_197896 | 0.0283 | 0.2567 | 0.0325 | 0.9998 |
| NM_001081236.1 | *2410131K14Rik* | Mus musculus RIKEN cDNA 2410131K14 gene (2410131K14Rik), mRNA. | 0.0067 | 0.2567 | 0.0326 | 0.9998 |
| AK032886 | *6720467J09Rik* | ILMN_204611 | 0.0365 | 0.2567 | 0.0327 | 0.9998 |
| NM_144826.3 | *Utp6* | Mus musculus UTP6, small subunit (SSU) processome component, homolog (yeast) (Utp6), mRNA. | 0.0362 | 0.2567 | 0.0331 | 0.9998 |
|  | *C430047I10Rik* | ILMN_190353 | 0.0395 | 0.2567 | 0.0332 | 0.9998 |
| XM_355347.1 | *LOC381383* | ILMN_197324 | 0.0367 | 0.2567 | 0.0332 | 0.9998 |
| NM_172419.1 | *9030612E09Rik* | ILMN_216819 | 0.0435 | 0.2569 | 0.0333 | 0.9998 |
| NM_010699.1 | *Ldha* | Mus musculus lactate dehydrogenase A (Ldha), mRNA. | 0.0234 | 0.2567 | 0.0334 | 0.9998 |
| XM_285674.2 | *LOC331066* | ILMN_199274 | 0.0021 | 0.2567 | 0.0336 | 0.9998 |
| NM_173404.2 | *Bmp3* | Mus musculus bone morphogenetic protein 3 (Bmp3), mRNA. | 0.0355 | 0.2567 | 0.0337 | 0.9998 |
| NM_146783.1 | *Olfr26* | Mus musculus olfactory receptor 26 (Olfr26), mRNA. | 0.0350 | 0.2567 | 0.0340 | 0.9998 |
| AK083478 | *Slc11a2* |  | 0.0367 | 0.2567 | 0.0340 | 0.9998 |
| AK017854 | *5730564E11Rik* | ILMN_201847 | 0.0452 | 0.2569 | 0.0341 | 0.9998 |
| XM_357784.1 | *LOC384675* | ILMN_200971 | 0.0248 | 0.2567 | 0.0341 | 0.9998 |
| AK014811 | *4921504I02Rik* | ILMN_202285 | 0.0165 | 0.2567 | 0.0343 | 0.9998 |
| XM_140032.2 | *LOC210143* | ILMN_198180 | 0.0161 | 0.2567 | 0.0343 | 0.9998 |
| XM_001477781.1 | *LOC100042550* | PREDICTED: Mus musculus similar to putative, transcript variant 1 (LOC100042550), mRNA. | 0.0375 | 0.2567 | 0.0344 | 0.9998 |
| XM_001479095.1 | *LOC100047902* | PREDICTED: Mus musculus similar to RIKEN cDNA 1700022C21 gene (LOC100047902), mRNA. | 0.0084 | 0.2567 | 0.0345 | 0.9998 |
| XM_001477976.1 | *LOC100047208* | PREDICTED: Mus musculus similar to trypsinogen 15 (LOC100047208), mRNA. | 0.0032 | 0.2567 | 0.0345 | 0.9998 |
| NM_011400.2 | *Slc2a1* | Mus musculus solute carrier family 2 (facilitated glucose transporter), member 1 (Slc2a1), mRNA. | 0.0066 | 0.2567 | 0.0347 | 0.9998 |
| NM_009491.2 | *V2r16* | Mus musculus vomeronasal 2, receptor, 16 (V2r16), mRNA. | 0.0016 | 0.2567 | 0.0347 | 0.9998 |
| XM_915050.2 | *Es31* | PREDICTED: Mus musculus esterase 31, transcript variant 5 (Es31), mRNA. | 0.0273 | 0.2567 | 0.0348 | 0.9998 |
| XM_001481328.1 | *Ddx10* | PREDICTED: Mus musculus DEAD (Asp-Glu-Ala-Asp) box polypeptide 10 (Ddx10), mRNA. | 0.0225 | 0.2567 | 0.0349 | 0.9998 |
| XM_137448.1 | *LOC237684* | ILMN_196850 | 0.0176 | 0.2567 | 0.0349 | 0.9998 |
| NM_025714.2 | *Odf2l* | Mus musculus outer dense fiber of sperm tails 2-like (Odf2l), mRNA. | 0.0354 | 0.2567 | 0.0349 | 0.9998 |
| NM_029545 | *6530401N04Rik* |  | 0.0304 | 0.2567 | 0.0350 | 0.9998 |
| NM_009338.3 | *Acat2* | Mus musculus acetyl-Coenzyme A acetyltransferase 2 (Acat2), mRNA. | 0.0066 | 0.2567 | 0.0351 | 0.9998 |
| XM_488680 | *E030007A22* | ILMN_215907 | 0.0397 | 0.2567 | 0.0351 | 0.9998 |
| XM_357793.1 | *LOC384689* | ILMN_200981 | 0.0144 | 0.2567 | 0.0352 | 0.9998 |
| XM_203753.2 | *Fvt1* | ILMN_218151 | 0.0028 | 0.2567 | 0.0353 | 0.9998 |
| XM_155903.2 | *LOC239770* | ILMN_197973 | 0.0282 | 0.2567 | 0.0356 | 0.9998 |
| NM_009322.3 | *Tbr1* | Mus musculus T-box brain gene 1 (Tbr1), mRNA. | 0.0221 | 0.2567 | 0.0358 | 0.9998 |
| AK043199 | *A730069A04Rik* | ILMN_205345 | 0.0276 | 0.2567 | 0.0362 | 0.9998 |
| NM_013637.4 | *Prm1* | Mus musculus protamine 1 (Prm1), mRNA. | 0.0415 | 0.2567 | 0.0363 | 0.9998 |
| XM_919429.2 | *4930527J03Rik* | PREDICTED: Mus musculus RIKEN cDNA 4930527J03 gene (4930527J03Rik), mRNA. | 0.0259 | 0.2567 | 0.0364 | 0.9998 |
| NM_013871.2 | *Mapk12* | Mus musculus mitogen-activated protein kinase 12 (Mapk12), mRNA. | 0.0289 | 0.2567 | 0.0364 | 0.9998 |
| NM_178629 | *4930579E17Rik* |  | 0.0253 | 0.2567 | 0.0365 | 0.9998 |
| NM_013772.1 | *Tcl1b3* | Mus musculus T-cell leukemia/lymphoma 1B, 3 (Tcl1b3), mRNA. | 0.0344 | 0.2567 | 0.0367 | 0.9998 |
| NM_001011869.1 | *Olfr452* | Mus musculus olfactory receptor 452 (Olfr452), mRNA. | 0.0254 | 0.2567 | 0.0367 | 0.9998 |
| NM_001033542.1 | *4933429E10Rik* | Mus musculus RIKEN cDNA 4933429E10 gene (4933429E10Rik), mRNA. | 0.0010 | 0.2567 | 0.0369 | 0.9998 |
| NM_025745.2 | *4933407N01Rik* |  | 0.0350 | 0.2567 | 0.0369 | 0.9998 |
| NM_007942.2 | *Epo* | Mus musculus erythropoietin (Epo), mRNA. | 0.0198 | 0.2567 | 0.0369 | 0.9998 |
| NM_026254.2 | *Tbc1d23* | Mus musculus TBC1 domain family, member 23 (Tbc1d23), mRNA. | 0.0252 | 0.2567 | 0.0375 | 0.9998 |
| XM_358121.1 | *LOC385212* | ILMN_201462 | 0.0238 | 0.2567 | 0.0375 | 0.9998 |
| NM_177036.2 | *Ceacam19* | Mus musculus CEA-related cell adhesion molecule 19 (Ceacam19), mRNA. | 0.0054 | 0.2567 | 0.0375 | 0.9998 |
| NM_001045540.1 | *OTTMUSG00000005523* | Mus musculus predicted gene, OTTMUSG00000005523 (OTTMUSG00000005523), mRNA. | 0.0240 | 0.2567 | 0.0378 | 0.9998 |
| AK044798 | *B130002B06Rik* | ILMN_204255 | 0.0125 | 0.2567 | 0.0383 | 0.9998 |
| NM_001037246.1 | *Il20rb* | Mus musculus interleukin 20 receptor beta (Il20rb), transcript variant 1, mRNA. | 0.0436 | 0.2569 | 0.0383 | 0.9998 |
| XM_001477013.1 | *Ptar1* | PREDICTED: Mus musculus protein prenyltransferase alpha subunit repeat containing 1 (Ptar1), mRNA. | 0.0138 | 0.2567 | 0.0384 | 0.9998 |
| NM_001001322.2 | *Adamts13* | Mus musculus a disintegrin-like and metallopeptidase (reprolysin type) with thrombospondin type 1 motif, 13 (Adamts13), mRNA. | 0.0408 | 0.2567 | 0.0385 | 0.9998 |
| NM_026431.2 | *1810043G02Rik* | Mus musculus RIKEN cDNA 1810043G02 gene (1810043G02Rik), mRNA. | 0.0121 | 0.2567 | 0.0385 | 0.9998 |
| AK080353 | *A630064D23Rik* | ILMN_206665 | 0.0174 | 0.2567 | 0.0390 | 0.9998 |
| AK030051 | *4932418B07Rik* | ILMN_205552 | 0.0195 | 0.2567 | 0.0391 | 0.9998 |
| XR_034206.1 | *LOC100048071* | PREDICTED: Mus musculus similar to tsec-2 (LOC100048071), misc RNA. | 0.0247 | 0.2567 | 0.0391 | 0.9998 |
| NM_011813.2 | *Fiz1* | Mus musculus Flt3 interacting zinc finger protein 1 (Fiz1), mRNA. | 0.0244 | 0.2567 | 0.0393 | 0.9998 |
| NM_010586.1 | *Itpr2* | Mus musculus inositol 1,4,5-triphosphate receptor 2 (Itpr2), transcript variant 2, mRNA. | 0.0295 | 0.2567 | 0.0394 | 0.9998 |
| NM_146381.1 | *Olfr1284* | Mus musculus olfactory receptor 1284 (Olfr1284), mRNA. | 0.0181 | 0.2567 | 0.0398 | 0.9998 |
| AK039072 | *A230092L08Rik* | ILMN_203584 | 0.0245 | 0.2567 | 0.0400 | 0.9998 |
| NM_032540.2 | *Kel* | Mus musculus Kell blood group (Kel), mRNA. | 0.0063 | 0.2567 | 0.0402 | 0.9998 |
| AK033509 | *Egln3* |  | 0.0106 | 0.2567 | 0.0405 | 0.9998 |
| XM_001481253.1 | *LOC669520* | PREDICTED: Mus musculus hypothetical LOC669520 (LOC669520), mRNA. | 0.0327 | 0.2567 | 0.0408 | 0.9998 |
| AK044331 | *Atp9b* |  | 0.0191 | 0.2567 | 0.0412 | 0.9998 |
| NM_009477.1 | *Upp1* | Mus musculus uridine phosphorylase 1 (Upp1), mRNA. | 0.0104 | 0.2567 | 0.0412 | 0.9998 |
| NM_183417.2 | *Cdk2* | Mus musculus cyclin-dependent kinase 2 (Cdk2), transcript variant 1, mRNA. | 0.0255 | 0.2567 | 0.0413 | 0.9998 |
| NM_021539.4 | *Wsb2* | Mus musculus WD repeat and SOCS box-containing 2 (Wsb2), mRNA. | 0.0264 | 0.2567 | 0.0413 | 0.9998 |
| NM_025861.2 | *Pqlc1* | Mus musculus PQ loop repeat containing 1 (Pqlc1), mRNA. | 0.0423 | 0.2569 | 0.0415 | 0.9998 |
| XM_001474081.1 | *LOC383196* | PREDICTED: Mus musculus hypothetical LOC383196 (LOC383196), mRNA. | 0.0142 | 0.2567 | 0.0418 | 0.9998 |
| NM_201375.1 | *Kng2* | Mus musculus kininogen 2 (Kng2), mRNA. | 0.0185 | 0.2567 | 0.0421 | 0.9998 |
| XM_146296.2 | *LOC234159* | ILMN_198869 | 0.0052 | 0.2567 | 0.0424 | 0.9998 |
| NM_025854.1 | *1700023B02Rik* | Mus musculus RIKEN cDNA 1700023B02 gene (1700023B02Rik), mRNA. | 0.0214 | 0.2567 | 0.0424 | 0.9998 |
| AK081911 | *C130086K02Rik* | ILMN_206217 | 0.0194 | 0.2567 | 0.0426 | 0.9998 |
| NM_025278.4 | *Gng12* | Mus musculus guanine nucleotide binding protein (G protein), gamma 12 (Gng12), mRNA. | 0.0201 | 0.2567 | 0.0439 | 0.9998 |
| AK087624 | *E230026L19Rik* | ILMN_206615 | 0.0173 | 0.2567 | 0.0440 | 0.9998 |
| NM_028990.3 | *Tmem168* | Mus musculus transmembrane protein 168 (Tmem168), mRNA. | 0.0285 | 0.2567 | 0.0440 | 0.9998 |
| XM_913690.2 | *Gpr39* | PREDICTED: Mus musculus G protein-coupled receptor 39 (Gpr39), mRNA. | 0.0468 | 0.2577 | 0.0440 | 0.9998 |
| XM_129558.5 | *EG226654* | PREDICTED: Mus musculus predicted gene, EG226654, transcript variant 1 (EG226654), mRNA. | 0.0033 | 0.2567 | 0.0442 | 0.9998 |
| AK076521 | *4833438J18Rik* | ILMN_207306 | 0.0202 | 0.2567 | 0.0443 | 0.9998 |
| XM_358739.1 | *LOC384285* | ILMN_200435 | 0.0477 | 0.2584 | 0.0444 | 0.9998 |
| NM_175219.3 | *C130026I21Rik* | Mus musculus RIKEN cDNA C130026I21 gene (C130026I21Rik), transcript variant 1, mRNA. | 0.0343 | 0.2567 | 0.0445 | 0.9998 |
| NM_026182.4 | *Mtfr1* | Mus musculus mitochondrial fission regulator 1 (Mtfr1), nuclear gene encoding mitochondrial protein, mRNA. | 0.0220 | 0.2567 | 0.0449 | 0.9998 |
| NM_016700 | *Mapk8* |  | 0.0355 | 0.2567 | 0.0450 | 0.9998 |
| NM_010250.4 | *Gabra1* | Mus musculus gamma-aminobutyric acid (GABA-A) receptor, subunit alpha 1 (Gabra1), mRNA. | 0.0229 | 0.2567 | 0.0450 | 0.9998 |
| NM_001040130.1 | *Tmem141* | Mus musculus transmembrane protein 141 (Tmem141), mRNA. XM_979169 | 0.0287 | 0.2567 | 0.0454 | 0.9998 |
| NM_172838.3 | *Slc16a12* | Mus musculus solute carrier family 16 (monocarboxylic acid transporters), member 12 (Slc16a12), mRNA. | 0.0262 | 0.2567 | 0.0455 | 0.9998 |
| NM_028034.2 | *Tdrd12* | Mus musculus tudor domain containing 12 (Tdrd12), transcript variant 2, mRNA. | 0.0255 | 0.2567 | 0.0459 | 0.9998 |
| AK045272 | *Msx2* |  | 0.0335 | 0.2567 | 0.0460 | 0.9998 |
| NM_053151.1 | *Klra21* | Mus musculus killer cell lectin-like receptor subfamily A, member 21 (Klra21), mRNA. | 0.0250 | 0.2567 | 0.0461 | 0.9998 |
| NM_001038587.1 | *Adar* | Mus musculus adenosine deaminase, RNA-specific (Adar), transcript variant 1, mRNA. | 0.0249 | 0.2567 | 0.0465 | 0.9998 |
| NM_001081318.1 | *EG625716* | Mus musculus predicted gene, EG625716 (EG625716), mRNA. | 0.0026 | 0.2567 | 0.0468 | 0.9998 |
| NM_175523.4 | *Ppm1k* | Mus musculus protein phosphatase 1K (PP2C domain containing) (Ppm1k), mRNA. | 0.0220 | 0.2567 | 0.0469 | 0.9998 |
| NM_011820.2 | *Ggtla1* | Mus musculus gamma-glutamyltransferase-like activity 1 (Ggtla1), mRNA. | 0.0164 | 0.2567 | 0.0470 | 0.9998 |
| NM_027976.2 | *Acsl5* | Mus musculus acyl-CoA synthetase long-chain family member 5 (Acsl5), mRNA. | 0.0200 | 0.2567 | 0.0471 | 0.9998 |
| AK019079.1 | *scl0003471.1_340* | ILMN_188747 | 0.0033 | 0.2567 | 0.0471 | 0.9998 |
| XM_141626.2 | *LOC245350* | ILMN_198536 | 0.0167 | 0.2567 | 0.0472 | 0.9998 |
| NM_007620.2 | *Cbr1* | Mus musculus carbonyl reductase 1 (Cbr1), mRNA. | 0.0319 | 0.2567 | 0.0473 | 0.9998 |
| NM_146422.1 | *Olfr767* | Mus musculus olfactory receptor 767 (Olfr767), mRNA. | 0.0434 | 0.2569 | 0.0475 | 0.9998 |
| NM_009863.2 | *Cdc7* | Mus musculus cell division cycle 7 (S. cerevisiae) (Cdc7), mRNA. | 0.0175 | 0.2567 | 0.0478 | 0.9998 |
| NM_028933.1 | *1300010M03Rik* | Mus musculus RIKEN cDNA 1300010M03 gene (1300010M03Rik), transcript variant 2, mRNA. | 0.0239 | 0.2567 | 0.0486 | 0.9998 |
| NM_017388.1 | *Ear3* | Mus musculus eosinophil-associated, ribonuclease A family, member 3 (Ear3), mRNA. | 0.0297 | 0.2567 | 0.0486 | 0.9998 |
| NM_001033788.1 | *BB287469* | Mus musculus expressed sequence BB287469 (BB287469), mRNA. | 0.0408 | 0.2567 | 0.0489 | 0.9998 |
| AK020408 | *Sast-pending* | ILMN_202605 | 0.0424 | 0.2569 | 0.0490 | 0.9998 |
| XM_193754.2 | *LOC268730* | ILMN_197620 | 0.0282 | 0.2567 | 0.0492 | 0.9998 |
| NM_019946.3 | *Mgst1* | Mus musculus microsomal glutathione S-transferase 1 (Mgst1), mRNA. | 0.0217 | 0.2567 | 0.0497 | 0.9998 |
| NM_025332.2 | *Gtpbp8* | Mus musculus GTP-binding protein 8 (putative) (Gtpbp8), mRNA. | 0.0352 | 0.2567 | 0.0500 | 0.9998 |
| NM_177005.2 | *Glt1d1* | Mus musculus glycosyltransferase 1 domain containing 1 (Glt1d1), mRNA. | 0.0456 | 0.2569 | 0.0500 | 0.9998 |

1. Association between gene expression and serum adiponectin concentration show no difference among the two dietary groups

| Accession | Symbol | Definition | P-value (interaction; M_PAG1_) | P-value (t-test; M_DEG_) | P-value (no interaction; M_PAG2_) | FDR  (no interaction) |
| --- | --- | --- | --- | --- | --- | --- |
| NM_019473.1 | *Olfr155* | Mus musculus olfactory receptor 155 (Olfr155), mRNA. | 0.0887 | 0.0415 | 0.0192 | 0.9997 |
| XR_035725.1 | *4930412O13Rik* | PREDICTED: Mus musculus RIKEN cDNA 4930412O13 gene (4930412O13Rik), misc RNA. | 0.1310 | 0.0295 | 0.0256 | 0.9997 |
| NM_198643.1 | *D630005B22Rik* | Mus musculus RIKEN cDNA D630005B22 gene (D630005B22Rik), mRNA. | 0.2332 | 0.0110 | 0.0310 | 0.9997 |

FDR, false discovery rate using a Benjamini and Hochberg multiple testing correction.
